# Impact of oral intervention on the oral and overall health of children living with HIV in Cambodia: a randomized controlled trial

**DOI:** 10.1186/s12916-023-02862-2

**Published:** 2023-04-28

**Authors:** Kimiyo Kikuchi, Sovannary Tuot, Junko Yasuoka, Makoto Murayama, Sumiyo Okawa, Akira Shibanuma, Keiko Nanishi, Sothearith Eng, Chantheany Huot, Siyan Yi

**Affiliations:** 1 Department of Health Sciences, Faculty of Medical Sciences, Kyushu University, Fukuoka, Japan; 2 KHANA Center for Population Health Research, Phnom Penh, Cambodia; 3 Department of Community and Global Health, Graduate School of Medicine, The University of Tokyo, Tokyo, Japan; 4 Faculty of Social Science and Humanity, Royal University of Phnom Penh, Phnom Penh, Cambodia; 5 Center for Infectious Disease Epidemiology and Prevention Research, Tokyo University of Agriculture and Technology, Tokyo, Japan; 6 Kawasaki City Dentists Association, Kanagawa, Japan; 7 Institute for Global Health Policy Research, Bureau of International Health Cooperation, National Center for Global Health and Medicine, Tokyo, Japan; 8 Office of International Academic Affairs, Graduate School of Medicine and Faculty of Medicine, The University of Tokyo, Tokyo, Japan; 9 Department of Global Public Health, Graduate School of Comprehensive Human Sciences, University of Tsukuba, Tsukuba, Japan; 10 National Pediatric Hospital, Phnom Penh, Cambodia; 11 Saw Swee Hock School of Public Health, National University of Singapore and National University Health System, Singapore, Singapore; 12 Center for Global Health Research, Touro University California, Vallejo, CA USA

**Keywords:** HIV, Child, Oral health, Randomized controlled trial, Cambodia

## Abstract

**Background:**

Maintaining oral health is essential for improving overall health of children living with HIV. Therefore, we evaluated the effectiveness of an oral health intervention for improving their oral and overall health. In addition, we examined their longitudinal association between changes in oral and overall health.

**Methods:**

We conducted a 2-year randomized controlled trial involving children living with HIV in Cambodia. Children aged 3–15 years and their caregivers were randomly allocated either to the intervention (group A) or control (group B) arm. A second control arm (group C) included children without HIV. The group A children received oral health education sessions and practiced home-based daily care.

**Results:**

In the baseline survey, 482 children participated (group A: *n* = 160, group B: *n* = 168, group C: *n* = 154), and 350 completed the endline survey. An interaction effect in teeth brushing duration was observed in children in group A relative to group B (AOR = 2.69, 95% CI: 1.37–5.31) and group C (AOR = 3.78, 95% CI: 1.70–8.40). Longitudinal associations were observed between changes in oral hygiene and overall health, as presented by alterations in dental caries in permanent teeth with viral load detection (adjusted odds ratio = 3.58, 95% CI: 1.10 − 11.73), in salivary flow quantity with the overall quality of life (*β* = 0.07, 95% CI: < 0.01 − 0.13), as well as in dental caries, salivary pH, debris index with body mass index for age among group A children.

**Conclusions:**

Oral health intervention may improve oral care behaviors and potentially enhance overall health among children living with HIV in antiretroviral therapy in a resource-constrained setting.

**Trial registration:**

ISRCTN 15177479.

**Supplementary Information:**

The online version contains supplementary material available at 10.1186/s12916-023-02862-2.

## Background

In 2021, 1.7 million children were living with HIV worldwide [1], only 52% of whom received antiretroviral therapy (ART). ART has reduced the mortality rate in children living with HIV by more than half over the past decade [2]. Moreover, early ART initiation can help children’s physical development more normally. Therefore, the treatment of this population should be essentially focused on healthy living and improving quality of life (QOL).

In children without HIV, oral health is associated with overall and long-term health. In general adult populations, poor oral health has been associated with chronic diseases, such as diabetes, respiratory and cardiovascular diseases, and cancer [3–6]. Previous studies showed that 42% of children aged 0–14 years experience oral disorders worldwide, with dental caries being the most common disorder [7]. Globally, 30% of children have dental caries in their deciduous teeth and 14% in their permanent teeth [7]. Furthermore, oral health has been closely correlated with overall health in children. A systematic review reported that children with dental caries were more likely to be overweight and obese [8]. Higher dental plaque index and salivary flow quantity, and decreased saliva production have been associated with stunting [9]. Therefore, oral health should be addressed in early childhood.

An association between oral and overall health indicators, such as viral load and QOL, has been observed in children living with HIV [10–12]. However, to the best of our knowledge, the effectiveness of oral health interventions in improving overall health in children living with HIV has not been well established yet. Furthermore, the differential impacts of the interventions among children living with and without HIV require further investigation. Children living with HIV exhibited a higher risk for poor oral and general health than their uninfected peers [13]. Hence, a comparison study is essential to assess the effect of interventions on the health status of children living with and without HIV.

In Cambodia, there has been a considerable decrease in the rates of new HIV infections and AIDS-related mortality in the last two decades [14]. This achievement can be attributed to the high coverage of ART, with 84% of people living with HIV receiving it in 2021 [14]. In Cambodia, 2,300 children were living with HIV in 2021 and < 100 new infections and AIDS-related deaths among children < 15 years old were reported in the same year [14]. Despite treatment successes, new infection and AIDS-related deaths were relatively higher than those of the neighboring countries in Southeast Asia [14].

The general children population in Cambodia carries a severe oral health burden caused by dental caries, with 57% of children aged 0–4 years having carious lesions [15]. Our previous study showed that children living with HIV were likely to exhibit worse oral health, as evidenced by dental caries and high salivary flow quantity, than children without HIV [13]. Under these circumstances, exploring effective interventions that can subsequently improve the overall health of children living with HIV is essential.

Therefore, this study aimed to evaluate the effectiveness of oral health interventions in improving the oral and overall health of children living with HIV receiving ART and compared the effects of the interventions between children with and without HIV. Furthermore, we examined the longitudinal association between oral and overall health changes.

## Methods

### Study design

This randomized controlled trial was conducted from February 2018 to April 2021 in the HIV clinic of the National Pediatric Hospital (NPH) in Phnom Penh, Cambodia. The original research plan included a 2-year intervention. However, owing to the coronavirus disease 2019 (COVID-19) pandemic, the endline study was postponed by 10 months. The study protocol has been published elsewhere [16].

### Participants

**Fig. 1:**
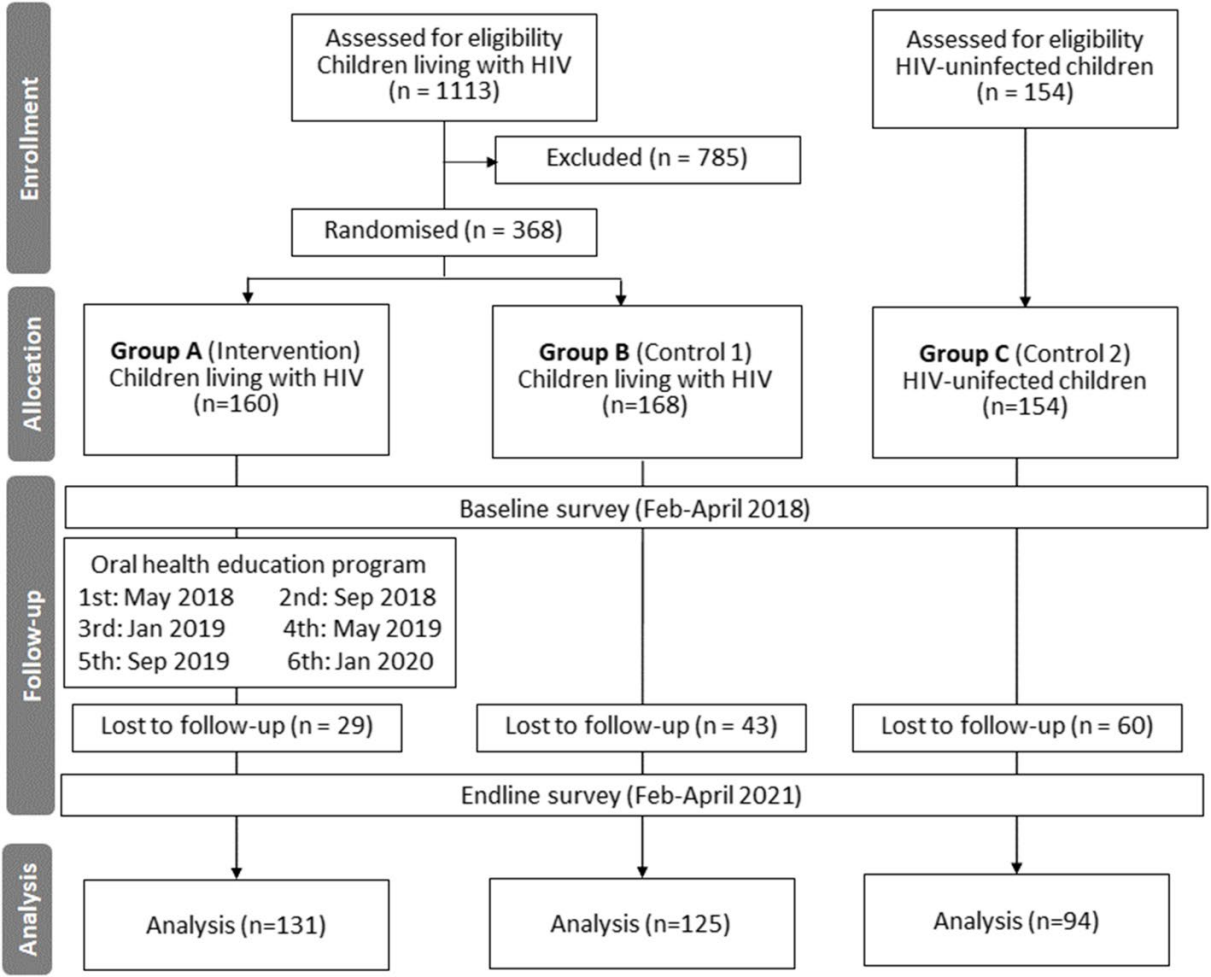
Flow diagram of the participants

The study population comprised children living with and without HIV. Of the 1113 eligible children in NPH, 482 were randomly included in the baseline survey (Fig. 1). Of the total, 160 and 168 children living with HIV were randomly assigned to the intervention (group A) and primary control (group B) arms, respectively. A second control arm was established, comprising 154 children without HIV (group C), to compare the oral health status of children living with HIV to that of children without HIV after the intervention. Moreover, primary caregivers of the children participated in the study as respondents in the baseline and endline surveys. The primary caregivers of group A attended regular oral health seminars alongside their children. The inclusion criteria for groups A and B were children aged 3–15 years at baseline and receiving HIV services at NPH at the baseline survey. Children in group C were in the same age group as those in groups A and B. In addition, they had not been diagnosed with HIV infection by the time of recruitment. The caregivers of all the children were eligible if they were the primary caregivers of the children and ≥ 18 years.

### Sample size

Before the intervention, the minimum required sample size was 260 per group based on the decayed, missing, and filled permanent teeth (DMFT) score obtained from other studies [16, 17]. However, the number of children living with HIV aged < 8 years was less owing to recent improvements in preventing mother-to-child transmission in Cambodia. As a result, the sample size was re-estimated based on the DMFT scores collected at baseline. Thereafter, the minimum required sample size was 160 per group. A post hoc power analysis was conducted according to the DMFT/dmft scores obtained from the baseline (8.0, SD: 4.8) and endline (5.0, SD: 3.8) surveys. This estimation provided a power of ≥ 80% with an alpha of 5% and a two-tailed test. Thus, the sample size used had sufficient power.

### Recruitment and randomization

Children living with HIV were identified from the patient list of the NPH HIV clinic. The participants were randomly assigned to either the intervention or control group, allowing equal allocation of same-aged children. A computerized algorithm was used to perform randomization by a data analyst who was not a primary member of the study team. Three districts in Phnom Penh had the highest number of children living with HIV who participated in this study. We randomly selected one village from each of the three districts and randomly recruited eligible children without HIV using the resident lists obtained from the village heads. Participants’ enrollment and intervention assignment were not concealed due to the nature of the intervention design.

### Interventions

The children in group A and their caregivers received oral health education sessions every four months, with a total of six sessions during the intervention period. During the sessions presentation materials and videos (e.g., how to prevent dental caries and what food is good or bad for the teeth) were used (Additional file 1: Appendix 1) to enable the participants to practice daily home-based oral health care (e.g., effective tooth brushing and flossing). We added explanations and practices that take into account the child’s development stage (involvement of caregivers in tooth brushing according to the child’s age, changes in caries risk according to age, etc.). At each session, we provided participants with a toothbrush, fluoride-containing toothpaste (sodium fluoride: 1450 ppm), and dental floss. We did not provide any intervention or materials to participants in groups B and C. No dental treatment or check-ups were provided to any children. However, they were allowed to go to the dentist of their free will.

### Outcome measures

The primary outcome associated with oral health was the change in DMFT or the decayed, missing, and filled deciduous teeth (dmft) scores, and the secondary outcome was the viral load. Initially, the CD4 count was considered the secondary outcome. However, after initiating the study, only viral load was used to measure disease progression in children living with HIV in Cambodia. Therefore, viral load was used as the study outcome, as shown in the revised online protocol (ISRCTN15177479). For oral health outcomes, we collected data regarding oral hygiene indicators (salivary pH, salivary flow quantity, debris index score, and oral health-related QOL [OHQOL]) and oral healthcare behavioral indicators (dental visits, brushing frequency and duration, and oral care support from caregivers). To measure the overall health outcomes, we collected information regarding height for age, body mass index (BMI) for age, and overall QOL in addition to viral load.

### Data collection

We conducted the baseline surveys from February to April 2018 and the endline survey from February to April 2021. The children and their caregivers were interviewed using a structured questionnaire. We also collected data on children’s oral health status, weight, and height. For children in groups A and B, data regarding viral load were also collected from the medical records of NPH. Additional file 1: Appendix 2 presents the details of the collected data.

#### Questionnaire

Trained research assistants interviewed children and caregivers. The questionnaires collected information regarding the sociodemographic, oral care behavior, overall health-related QOL, and OHQOL.

Regarding oral healthcare behaviors, the participants were asked whether they had visited a dentist over the last 12 months owing to pain or other problems related to teeth, their frequency of brushing per day, duration of teeth brushing per occasion, and whether their caregiver had ever helped them with teeth brushing.

The overall health-related QOL was evaluated using the Pediatric Quality of Life Inventory (PedsQL™ 4.0) [18], which included 23 questions. The total score ranges from 0 to 100, with a higher score indicating higher overall health-related QOL. The OHQOL was assessed using the Child Perceptions Questionnaire 11–14 [19], which includes 16 questions with scores ranging from 0 to 64. We used it for children ≥ 6 years old because it was validated in the previous studies in Cambodia and New Zealand [19, 20]. A higher score indicates a lower OHQOL.

#### Registered medical records

Data regarding viral load were collected from the registered medical records of the NPH by research assistants. The latest information regarding viral load was obtained before the baseline survey and after the endline survey.

#### Oral status examination

To evaluate children’s oral health status, we collected data regarding the number of DMFT/dmft, debris index, salivary pH, and salivary flow rate. A lower DMFT/dmft score and debris index, and a higher salivary flow rate and salivary pH indicated better oral health status. The dentists of NPH collected the complete oral health data with the help of dental assistants based on the WHO guideline [21]. The reproducibility of the intra- and inter-examiner ratings was evaluated. The dentists performed DMFT/dmft examinations on 10 children and made intergroup comparisons. The agreement rate of the results was ≥ 0.85. The debris index was calculated based on the amount of dental plaque on the dental surface. Data regarding salivary pH were collected using the salivary test kit (CAT21 Buf; Morita, Japan), which records pH values from 4.0 to 6.5. The total saliva quantity was calculated using the salivary flow data obtained after three minutes of tasteless chewing gum stimulation.

#### Body weight and height

Data regarding children’s body weight and height were collected and converted to the *Z*-scores of height and BMI for age. The conversions were performed using WHO AnthroPlus (available at https://www.who.int/tools/growth-reference-data-for-5to19-years/application-tools).

### Statistical analyses

First, we conducted descriptive analyses of the basic characteristics of the participants. The outcomes were examined for data with normal distribution, and skewed data were log-transformed. Second, bivariate analyses were performed to identify differences in variables in the baseline survey between groups A and B and between groups A and C to evaluate the efficacy of the intervention in improving oral and overall health outcomes. Third, we conducted mixed-model analyses between groups A and B and between groups A and C. The model was adjusted for age, sex, intervention type (intervention or control), time (baseline or endline), and interaction between intervention type and time. Fourth, mixed-model analyses of group A were performed to assess the longitudinal association between changes in oral and overall health outcomes. In addition to oral health outcomes, the model was adjusted for age, sex, and time (baseline or endline). Each oral health outcome was assessed separately with the overall health outcomes to prevent multicollinearity. Oral health outcomes were examined for association with overall health outcomes with continuous variables, while the association with viral load was examined with a binary variable with the mean as the cutoff. We also stratified children’s dentition types at the baseline (mixed dentition and permanent teeth dentition) and performed above mentioned mixed model analyses. We did not conduct stratified analyses on the deciduous teeth dentition as the sample size was not large enough. The threshold for statistical significance was *p*-value < 0.05. All data analyses were performed using the SPSS software version 28.0 (SPSS Inc., Chicago, IL, the USA). Sensitivity analyses were conducted in the third and fourth steps with the generalized estimating equation model adjusted using the same variables as in the mixed-model analyses.

## Results

### Retention rate at the endline survey

We started recruiting baseline survey participants in February 2018 and followed up with them until April 2021. The mean attendance of group A in six oral health education sessions was 83.3%. Finally, 86.3% (*n* = 138) completed the endline survey. In group B, 74.4% (*n* = 125) of the participants completed the endline survey. The reasons behind the loss to follow-up included refusal to participate, loss of contact, moving out, death, and transfer of clinic or residence. In group C, 154 participants were selected for the study, 61.0% (*n* = 94) of whom completed the endline survey. Most participants of group C who did not join the endline survey had moved to pursue higher education elsewhere.

### Characteristics of the participants

Table 1 shows the baseline characteristics of the participants, and Additional file 1: Appendix 3 shows the characteristics of the participants in the endline survey. Additional file 1: Appendix 4 shows a secondary table presenting data regarding the baseline, endline survey, and follow-up loss. At baseline, dental visits because of pain or problems with teeth and the family wealth index between groups A and B significantly differed. At baseline, groups A and C exhibited significant differences in dentition type, permanent teeth, salivary flow quantity, dental visits because of pain or tooth problems, caregivers’ support during tooth brushing, and family wealth index.

**Table 1:** Baseline characteristics of the participants

Characteristics	All participants (*n* = 481)		Group A Intervention group (HIV-positive, *n* = 160)		Group B Control group (HIV-positive, *n* = 168)		Group C Control group (HIV-negative, *n* = 154)		Difference between groups A and B, *P*-value**	Difference between groups A and C, *P*-value**
Child’s sex, female, *n* (%)	222	46.1	78	51.3	80	45.5	64	41.6	0.28	0.09
Child’s age, mean (SD)	10.8	3.0	10.7	3.1	10.9	2.9	10.6	2.9	0.75	0.59
< 6 years old, *n* (%)	30	6.2	7	4.4	9	5.4	11	7.1		
6–8 years old, *n* (%)	83	17.3	30	18.8	27	16.1	26	16.9		
9–12 years old, *n* (%)	215	44.7	65	40.6	78	46.4	72	46.8		
13–15 years old, *n* (%)	154	32.0	55	34.4	54	32.1	45.0	29.2		
Dentition type									0.74	0.02
Permanent teeth, *n* (%)	189	39.2	71	44.4	69	41.1	49	31.8		
Mixed dentition,* n* (%)	263	54.6	77	48.1	88	52.4	98	63.6		
Deciduous teeth,* n* (%)	30	6.2	12	7.5	11	6.5	7	4.5		
Child’s overall health status										
Viral load detected^a^, *n* (%)	104	31.7	47	30.9	57	32.4	n/a	n/a	0.86	n/a
Height for age in *z*-score, mean (SD)	− 2.0	1.4	− 2.0	1.4	− 1.8	1.4	− 2.2	1.4	0.51	0.09
BMI for age in *z*-score, mean (SD)	− 0.9	1.2	− 1.1	1.1	− 0.9	1.2	− 0.7	1.4	0.59	0.10
Overall health-related quality of life, mean (SD)	80.0	10.5	79.8	11.6	80.1	10.2	80.2	9.7	0.90	0.49
Child’s oral hygiene status										
Oral health-related quality of life, mean (SD)	12.0	7.3	11.8	7.6	11.9	7.2	12.2	7.1	0.90	0.62
Decayed, missing, filled teeth, mean (SD)**										
DMFT/dmft (total teeth) (*n* = 481)	8.0	4.8	8.1	4.8	7.6	5.1	8.2	4.7	0.70	0.23
Permanent teeth (*n* = 451)	3.9	3.7	4.0	3.6	4.2	3.7	3.4	3.8	0.71	0.27
Deciduous teeth (*n* = 292)	7.1	4.8	7.6	4.6	6.5	5.1	7.3	4.4	0.08	0.46
Salivary pH, mean (SD)	6.1	0.5	6.0	0.6	6.0	0.6	6.2	0.4	0.35	0.04
Salivary flow quantity/3 min, mean (SD)	2.7	1.3	2.9	1.3	2.8	1.3	2.2	1.3	0.39	< 0.01
Debris index, mean (SD)	2.2	0.7	2.2	0.7	2.1	0.7	2.4	0.7	0.62	0.09
Oral mucosal lesions										
Have oral mucosal lesions	63	13.1	26	16.3	27	16.1	n/a	n/a	0.97	n/a
Abscess	54	11.2	26	16.3	23	13.7	n/a	n/a		
Ulceration	5	1.0	1	0.6	3	1.8	n/a	n/a		
Acute necrotizing ulcerative gingivitis	4	0.8	0	0	1	0	n/a	n/a		
Child’s oral care behavior										
Dental visit within the last 12 months due to pain or trouble with teeth (n = 451), *n* (%)	99	22.0	41	29.1	34	20.4	24	16.8	0.03	0.01
Frequency of tooth brushing per day (*n* = 452), *n* (%)									0.15	0.05
Three times or more	115	25.4	41	27.5	40	25.0	34	23.8		
Twice a day	133	29.4	47	31.5	48	30.0	38	26.6		
Once a day or less	93	20.5	34	22.8	38	23.8	21	14.7		
Never clean teeth	111	24.6	27	18.1	34	21.3	50	35.0		
Duration of tooth brushing (*n* = 424), *n* (%)									0.44	0.09
≤ 1 min	118	27.8	48	36.7	40	25.4	30	22.2		
2 min	99	23.3	25	19.1	37	23.4	37	27.4		
≥ 3 min	207	48.8	58	44.3	81	51.3	68	50.3		
Caregivers’ support in tooth brushing (*n* = 439), *n* (%)									0.69	0.04
Caregivers brushed the children’s teeth or watched/advised while children were brushing	27	6.1	13	9.6	11	6.1	3	2.2		
Caregivers never cared about tooth brushing	412	93.8	123	90.4	153	93.3	136	97.8		
Family wealth index, *n* (%)									0.01	0.01
Lowest	95	19.8	28	17.5	52	31.0	15	9.9		
Highest	93	19.4	41	25.6	21	12.5	31	20.4		

^a^Children in group C were excluded from the total number of participants

^**^Continuous variables were log transformed. Mann–Whitney *U* test was used to evaluate nonparametric continuous variables, and the chi-square test was utilized to assess categorical variables

*Abbreviations*: *BMI* body mass index, *HIV* human immunodeficiency virus, *SD* standard deviation

As shown in Additional file 1: Appendix 3, the total DMFT/dmft of groups A, B, and C at the endline survey were 4.1 (SD 3.4)/4.9 (SD 3.6), 4.0 (SD 3.4)/4.7 (SD 4.4), and 2.6 (SD 3.0)/4.7 (SD 4.5), respectively. Those with a detected viral load in groups A and B at the endline survey were 16.2% and 14.4%, respectively.

### Effects of the intervention on oral and overall health outcomes among children living with HIV

Table 2 shows the intervention effects between groups A and B via mixed-model analyses stratified by dentition type at the baseline survey. In any of the dentition types, no interaction effect was identified in the oral hygiene outcomes, including OHQOL, salivary pH and flow quantity, DMFT/dmft scores, and debris index. However, a positive interaction effect was observed in the oral health care outcomes, particularly brushing for three or more times per day among all children (adjusted odds ratio [AOR] = 2.06, 95% CI: 1.03–4.12, *p* = 0.03) and children with permanent dentition (AOR = 3.72, 95% CI: 1.40–9.93, *p* = 0.01), as well as for ≥ 3 min per occasion among all children (AOR = 2.69, 95% CI: 1.37–5.31, *p* =  < 0.01) and children with permanent dentition (AOR = 4.53, 95% CI: 1.56–13.20, *p* = 0.01). No difference was observed in dental visits because of pain or problems with teeth, and caregivers’ support during tooth brushing.

**Table 2:** Effect of the intervention on oral and overall health outcomes between the intervention and control groups of children living with HIV (all dentitions, mixed dentition, permanent teeth dentition types at baseline)

Variables	Intervention × time																
	All dentition types at baseline					Mixed dentition at baseline							Permanent teeth dentition at baseline				
	Estimate	95% CI		*P* value		Estimate			95% CI		*P* value		Estimate	95% CI		*P* value	
Viral load detected (n, %)																	
Baseline																	
Endline	1.25	0.58	2.69	0.56		1.04		0.41		2.69	0.93		2.17	0.47	9.95	0.32	
Height for age (mean, SD)																	
Baseline																	
Endline	0.01	− 0.01	0.03		0.32	0.01		− 0.02		0.03	0.65		< − 0.01	− 0.02	0.02	0.83	
Body mass index for age (mean, SD)																	
Baseline																	
Endline	− 0.02	− 0.05	0.01		0.15	− 0.02		− 0.06		0.02	0.37		− 0.02	− 0.05	0.01	0.24	
Overall QOL (mean, SD)																	
Baseline																	
Endline	< 0.01	− 0.02	0.02		0.69	0.01		− 0.01		0.04	0.31		− 0.01	− 0.03	0.02	0.71	
OHQOL (mean, SD)																	
Baseline																	
Endline	0.01	− 0.04	0.05		0.77	− 0.02		− 0.09		0.05	0.55		0.03	− 0.02	0.09	0.25	
Salivary pH (mean, SD)																	
Baseline																	
Endline	< − 0.01	− 0.01	0.01		0.73	− 0.01		− 0.03		0.01	0.29		0.01	− 0.01	0.02	0.58	
Salivary flow (mean, SD)																	
Baseline																	
Endline	− 0.03	− 0.09	0.03		0.38	− 0.02		− 0.11		0.06	0.57		− 0.01	− 0.10	0.09	0.90	
DMFT/dmft (total teeth) (mean, SD)																	
Baseline																	
Endline	0.04	− 0.03	0.11		0.27		0.08	− 0.02		0.17	0.12	0.01		− 0.09	0.11		0.84
DMFT (permanent teeth) (mean, SD)																	
Baseline																	
Endline	0.05	− 0.02	0.12		0.14		0.07	− 0.03		0.18	0.16	0.02		− 0.08	0.11		0.75
dmft (deciduous teeth) (mean, SD)																	
Baseline																	
Endline	− 0.03	− 0.17	0.12		0.70		0.06	− 1.80		1.92	0.95	n/a		n/a	n/a		n/a
Debris index (mean, SD)																	
Baseline																	
Endline	0.01	− 0.03	0.05		0.63		0.05	0.00		0.10	0.07	− 0.02		− 0.08	0.04		0.52
Visited a dentist for pain or trouble with teeth (*n*, %)																	
Baseline																	
Endline	0.48	0.21	1.12		0.09		0.62	0.20		1.93	0.40	0.24		0.06	1.01		0.05
Brush three times and more per day (*n*, %)																	
Baseline																	
Endline	2.06	1.03	4.12		0.03*		1.19	0.43		3.33	0.74	3.72		1.40	9.93		0.01*
Brush for three minutes or more (*n*, %)																	
Baseline																	
Endline	2.69	1.37	5.31		< 0.01*		1.88	0.74		4.79	0.18	4.53		1.56	13.20		0.01*
Caregiver never care child’s toothbrushing (*n*, %)																	
Baseline																	
Endline	0.51	0.13	1.93		0.32		0.53	0.10		2.76	0.44	0.29		0.02	5.31		0.40

Adjusting with intervention (intervention or control), time (baseline or endline), and interaction of intervention and time, age, sex

*OHQOL* oral health-related quality of life, *DMFT* decayed, missing filled permanent teeth, *dmft* decayed, missing filled deciduous teeth

^*^p-value significant at < 0.05

### Effects of the intervention on oral and overall health outcomes in children with and without HIV

Table 3 shows the effect of the intervention on children with and without HIV (groups A and C) stratified by dentition types at the baseline survey. Among all children, an interaction effect was identified in height for age (*β* =  − 0.06, 95% CI: − 0.08 to − 0.04, *p* =  < 0.01), salivary flow quantity (*β* =  − 0.20, 95% CI: − 0.27 to − 0.13, *p* =  < 0.01), and DMFT/dmft scores (*β* = 0.23, 95% CI: 0.16–0.31, *p* =  < 0.01). Among children with mixed dentition, an interaction effect was identified in height for age, and among children with permanent teeth dentition, an interaction effect was identified in height for age, salivary flow, and DMFT/dmft score.

**Table 3:** Effectiveness of the intervention in improving oral and overall health outcomes between children living with HIV and without HIV (all dentitions, mixed dentition, permanent teeth dentition types at baseline)

Variables	Intervention × time															
	All dentition types at baseline					Mixed dentition at baseline						Permanent teeth dentition at baseline				
	Estimate	95% CI		*P* value		Estimate		95% CI			*P* value	Estimate	95% CI		*P* value	
Height for age (mean, SD)																
Baseline																
Endline	− 0.06	− 0.08	− 0.04	< 0.01*		− 0.06		− 0.09	− 0.03		< 0.01*	− 0.05	− 0.08	− 0.02	< 0.01*	
Body mass index for age (mean, SD)																
Baseline																
Endline	0.03	< 0.01	0.07		0.05	0.03	− 0.03		0.09	0.35		0.03	− 0.02	0.07		0.26
Overall QOL (mean, SD)																
Baseline																
Endline	0.01	− 0.01	0.03		0.51	0.02	− 0.01		0.04	0.23		− 0.01	− 0.04	0.03		0.73
OHQOL (mean, SD)																
Baseline																
Endline	< 0.01	− 0.05	0.06		0.92	− 0.02	− 0.09		0.05	0.55		0.02	− 0.06	0.11		0.58
Salivary pH (mean, SD)																
Baseline																
Endline	< 0.01	− 0.01	0.02		0.51	< 0.01	− 0.01		0.02	0.78		< 0.01	− 0.02	0.03		0.77
Salivary flow (mean, SD)																
Baseline																
Endline	− 0.20	− 0.27	− 0.13		< 0.01*	− 0.21	− 0.31		− 0.11	< 0.01		− 0.14	− 0.26	− 0.02		0.02*
Total teeth (mean, SD)																
Baseline																
Endline	0.23	0.16	0.31		< 0.01*	0.18	0.09		0.28	< 0.01		0.16	0.03	0.28		0.02*
DMFT (permanent teeth) (mean, SD)																
Baseline																
Endline	0.08	< 0.01	0.16		0.05	0.08	− 0.03		0.19	0.13		0.16	0.03	0.28		0.02*
dmft (deciduous teeth) (mean, SD)																
Baseline																
Endline	0.10	− 0.04	0.24		0.18	0.10	− 0.04		0.24	0.17		n/a	n/a	n/a		n/a
Debris index (mean, SD)																
Baseline																
Endline	− 0.01	− 0.05	0.04		0.78	0.03	− 0.02		0.08	0.30		− 0.01	− 0.08	0.07		0.87
Visited a dentist for pain or trouble with teeth (*n*, %)																
Baseline																
Endline	0.24	0.10	0.57		< 0.01*	0.30	0.09		0.98	0.05		0.20	0.03	1.30		0.09
Brush three times and more per day (*n*, %)																
Baseline																
Endline	1.96	0.88	4.36		0.10	1.81	0.62		5.31	0.28		3.26	0.67	15.85		0.14
Brush for three minutes or more (*n*, %)																
Baseline																
Endline	3.78	1.70	8.40		0.01*	2.74	0.96		7.80	0.06		0.54	1.19	24.56		0.03*
Caregiver never care child’s toothbrushing (*n*, %)																
Baseline																
Endline	3.24	0.56	18.70		0.19	3.20	0.48		21.18	0.23		n/a	n/a	n/a		n/a

Adjusting with intervention (intervention or control), time (baseline or endline), and interaction of intervention and time, age, sex

*OHQOL* oral health-related quality of life, *DMFT* decayed, missing filled permanent teeth, *dmft* decayed, missing filled deciduous teeth

^*^p-value significant at < 0.05

Regarding the oral health care outcomes, among all children, an interaction effect was identified in dental visits because of pain or problems with teeth (AOR = 0.24, 95% CI: 0.10–0.57, *p* =  < 0.01) and brushing for ≥ 3 min per occasion (AOR = 3.78, 95% CI: 1.70–8.40, *p* = 0.01). Those two interaction effects were also identified among children with permanent dentition. However, no significant difference was observed in tooth brushing frequency and caregivers’ support provided during tooth brushing.

### Longitudinal association oral and overall health changes in the intervention group

Table 4 depicts the longitudinal association between oral health outcomes and subsequent changes in overall health in the intervention arm (group A). Among all children, the changes in salivary pH exhibited an interaction effect on height for age (*β* =  − 0.57, 95% CI: − 0.92 to − 0.21, *p* < 0.01) and BMI for age (β = 0.71, 95% CI: 0.20–1.23, *p* = 0.01). The changes in salivary flow exhibited a positive interaction effect on overall QOL (*β* = 0.07, 95% CI: 0.01 − 0.13, *p* = 0.03). The changes in total of DMFT/dmft scores (*β* =  − 0.07, 95% CI: − 0.14 to − 0.01, *p* = 0.03) and debris index (*β* =  − 0.20, 95% CI: − 0.36 to − 0.04, *p* = 0.02) demonstrated negative interaction effects on BMI for age.

**Table 4:** Longitudinal association between oral health outcomes and overall health outcomes changes in the intervention group

Variables	Viral load detected^a^				Height for age				Body mass index for age				Overall QOL			
	**Exp**	**95% CI**		***P*-value**	**Estimate**	**95% CI**		***P*-value**	**Estimate**	**95% CI**		***P*-value**	**Estimate**	**95% CI**		***P*-value**
OHQOL	0.62	0.28	1.38	0.25	− 0.05	− 0.12	0.03	0.23	0.05	− 0.05	0.16	0.97	− 0.22	− 0.28	− 0.16	< 0.01
Time	0.61	0.27	1.41	0.25	− 0.01	− 0.14	0.12	0.85	− 0.44	− 0.63	− 0.25	< 0.01	− 0.04	− 0.15	0.07	0.48
OHQOL × time	0.98	0.29	3.31	0.97	0.02	− 0.08	0.12	0.69	0.07	− 0.08	0.22	0.34	0.05	− 0.04	0.13	0.28
Salivary pH	1.38	0.65	2.90	0.40	0.38	0.11	0.64	0.01	0.11	− 0.26	0.48	0.57	− 0.03	− 0.26	0.20	0.79
Time	0.74	0.33	1.66	0.46	0.46	0.18	0.74	< 0.01	− 0.91	− 1.31	− 0.51	< 0.01	0.13	− 0.14	0.39	0.36
Salivary pH × time	0.52	0.16	1.67	0.27	− 0.57	− 0.92	− 0.21	< 0.01	0.71	0.20	1.23	0.01	− 0.11	− 0.45	0.23	0.52
Salivary flow	0.91	0.41	2.01	0.81	0.02	− 0.03	0.07	0.40	0.04	− 0.03	0.11	0.26	− 0.06	− 0.11	− 0.02	0.01
Time	0.49	0.23	1.07	0.07	0.03	− 0.01	0.06	0.13	− 0.39	− 0.44	− 0.34	< 0.01	0.01	− 0.03	0.04	0.73
Salivary flow × time	1.25	0.40	3.94	0.71	− 0.02	− 0.09	0.04	0.48	0.09	− 0.01	0.18	0.08	0.07	0.01	0.13	0.03
DMFT/dmft	0.59	0.25	1.37	0.22	− 0.02	− 0.06	0.02	0.44	− 0.03	− 0.08	0.03	0.31	< − 0.01	− 0.04	0.03	0.68
Time	0.64	0.31	1.33	0.23	0.02	− 0.02	0.05	0.42	− 0.30	− 0.35	− 0.24	< 0.01	0.04	< − 0.01	0.08	0.05
DMFT/dmft × time	0.75	0.23	2.46	0.63	< 0.01	− 0.04	0.04	0.98	− 0.07	− 0.14	− 0.01	0.03*	< − 0.01	− 0.05	0.04	0.93
DMFT	0.71	0.29	1.78	0.47	− 0.01	− 0.05	0.03	0.66	− 0.01	− 0.06	0.05	0.83	0.14	− 0.02	0.05	0.40
Time	0.31	0.14	0.68	< 0.01	0.01	− 0.02	0.04	0.41	− 0.32	− 0.36	− 0.27	< 0.01	0.04	0.01	0.07	0.01
DMFT × time	3.58	1.10	11.73	0.04	< 0.01	− 0.04	0.05	0.85	− 0.06	− 0.12	< 0.01	0.06	− 0.01	− 0.05	0.03	0.67
Debris index	1.57	0.69	3.61	0.28	− 0.05	− 0.15	0.05	0.32	− 0.04	− 0.18	0.10	0.55	< 0.01	− 0.09	0.09	0.92
Time	0.70	0.31	1.58	0.39	0.01	− 0.04	0.06	0.82	− 0.31	− 0.38	− 0.24	< 0.01	0.03	− 0.02	0.08	0.25
Debris index × time	0.86	0.16	4.52	0.86	< 0.01	− 0.12	0.12	1.00	− 0.20	− 0.36	− 0.04	0.02*	0.04	− 0.07	0.15	0.46

Adjusted for age, sex, intervention, time (baseline or endline), interaction of each variable and time

*Abbreviations*: *CI* confidence interval, *DMFT* decayed, missing filled teeth, *OHQOL* oral health-related quality of life, *QOL* quality of life

The reference of the time variable was set as endline = 1 and baseline = 0

^a^Binary values with a mean cut off was used for the analysis: above mean = 1, below mean = 0

The aggravation of the DMFT score exhibited a positive interaction effect on viral load detection (AOR = 3.58, 95% CI: 1.10–11.73, *p* = 0.04).

Additional file 1: Appendix 8 shows the longitudinal associations among other dentition types. Among the children with mixed dentition, the changes in salivary pH exhibited an interaction effect on height for age, and DMFT/dmft exhibited an interaction effect on BMI for age. Among the children with permanent dentition, salivary pH and salivary flow exhibited an interaction effect on BMI for age.

## Discussion

To the best of our knowledge, this was the first randomized controlled trial that evaluated the effectiveness of oral health interventions in improving oral health outcomes among children living with HIV. We compared outcome indicators between children living with HIV receiving and not receiving the intervention and between children living with HIV receiving the intervention and children without HIV not receiving any intervention.

Oral health intervention improved the oral health care behaviors of children living with HIV compared to the control groups’ children. However, the differences in oral hygiene did not significantly differ between them. Those were similar even after stratification by mixed or permanent dentition type. The results are consistent with previous systematic reviews and meta-analyses of intervention studies involving the general child and adolescent populations [22–24]. The lack of significant difference in oral hygiene can be partially attributable to the short intervention duration of the present study. Previous studies identified oral care behavior changes in a relatively short period (2 weeks to 9 months) from oral health education programs [22–24]. Interventions shorter than 2 years are unlikely to significantly improve oral hygiene, such as DMFT scores [25]. Another meta-analysis revealed insufficient evidence regarding the effectiveness of oral health education programs in reducing dental caries, except for an intervention implemented for > 4 years [22]. Herein, oral health care education lasted 21 months, and the follow-up period was 2 years. Whether the duration of our study was sufficient for assessing changes in oral care behaviors remained uncertain. However, it was likely not adequate to assess changes in oral hygiene.

We found that children without HIV exhibited better oral hygiene, as assessed via the DMFT/dmft scores and salivary flow rate, than children living with HIV receiving the intervention. A similar association was observed in our cross-sectional analysis using this trial’s baseline data [13]. This finding indicates that HIV infection negatively impacts the oral health of children living with HIV. Therefore, our oral health care approach might be insufficient to reduce the difference in oral health status among the comparison groups, despite improved oral health care practice in children in the intervention arm. Therefore, children living with HIV are more likely to require strengthened oral health care than children without HIV. Further extended interventions using different approaches beyond primary oral health care education are warranted.

Longitudinal changes in oral hygiene outcomes due to the intervention were associated with changes in viral load and BMI for age in children living with HIV. Specifically, greater dental caries in permanent teeth was associated with viral load detection. Although this relationship has been known in a cross-sectional study [11], the results of this study suggested it in a longitudinal association too. Furthermore, decreased dental caries and dental plaque and increased salivary pH were associated with increased BMI for age. Such associations were identified in several cross-sectional studies of children without HIV [26–28]. This finding indicates that dental caries and toothache caused by chronic poor oral hygiene may lead to chewing difficulties and appetite loss, thereby affecting BMI [29]. Identifying factors associated with improved BMI for age in children living with HIV who are likely to have a generally lower BMI for age than those without HIV is critical. Additionally, increased salivary flow quantity was associated with increased QOL, physiological functions, such as tasting and swallowing, and cleaning effect for better oral hygiene [30]. The salivary flow might impact QOL, as reported in a study of adolescents with diabetes where those with xerostomia exhibited low QOL [31].

This study had certain limitations. The endline survey was delayed for 10 months because of the COVID-19 pandemic. The time gap and COVID-19 prevention and intervention measures might have influenced the direct impact of the intervention (e.g., oral healthcare behaviors, including oral hygiene for preventing infections, and interruptions in dental services in the study site and community). The children without HIV who dropped out from the study during the follow-up were older and moved elsewhere for higher schooling. Older children are likely to have higher DMFT scores in permanent teeth and better oral hygiene care skills, which might have affected our results. Despite these limitations, our study demonstrated a robust design involving children living with and without HIV recruited from a large health facility and comparable community. Hence, our results have critical implications for HIV care services for children and research on oral hygiene, development, and overall health.

## Conclusions

Oral health education improved the oral care behaviors of children living with HIV in a resource-constrained setting. In addition, over time, improved oral hygiene outcomes were associated with improved overall health outcomes, such as viral load, BMI for age, and QOL, among children living with HIV. Therefore, oral interventions may be provided to improve the overall health of children living with HIV in low- and middle-income countries.

## Supplementary Information

Additional file 1: Appendix 1. Training Schedule. Appendix 2. Outcome measures. Appendix 3. Participants’ characteristics at the baseline and endline surveys. Appendix 4. Followed-up and lost-to-follow up participants. Appendix 5. Differences of baseline and endline oral hygiene status in each participant group. Appendix 6. Age group stratified effect of the intervention on oral and overall health outcomes between the intervention and control groups of children living with HIV. Appendix 7. Age group stratified effectiveness of the intervention in improving oral and overall health outcomes between children living with HIV and without HIV. Appendix 8. Dentition type stratified longitudinal association between oral health outcomes and overall health outcomes changes in the intervention group. Appendix 9. Age group stratified longitudinal association between oral health outcomes and overall health outcomes changes in the intervention group.

## Data Availability

The data that support the findings of this study are available from the corresponding author, upon reasonable request.

## Abbreviations

AIDS: Acquired immune deficiency syndrome
AOR: Adjusted odds ratio
ART: Antiretroviral therapy
BMI: Body mass index
CI: Confidence interval
COVID-19: Coronavirus disease 2019
DMFT: Decayed, missing, and filled permanent teeth
dmft: Decayed, missing, and filled deciduous teeth
HIV: Human immunodeficiency virus
NPH: National Pediatric Hospital
OHQOL: Oral health-related quality of life
PedsQL: Pediatric Quality of Life Inventory
QOL: Quality of life
SD: Standard deviation
WHO: World Health Organization
