# Supplementary material for: Impact of oral intervention on the oral and overall health of children living with HIV in Cambodia: a randomized controlled trial
Source: BMC Med. 2023 Apr 28;21:162. doi: 10.1186/s12916-023-02862-2 (PMC10144884; doi:10.1186/s12916-023-02862-2)
Supplement: Supplementary file 1 — Additional file 1: Appendix 1. Training Schedule. Appendix 2. Outcome measures. Appendix 3. Participants’ characteristics at the baseline and endline surveys. Appendix 4. Followed-up and lost-to-follow up participants. Appendix 5. Differences of baseline and endline oral hygiene status in each participant group. Appendix 6. Age group stratified effect of the intervention on oral and overall health outcomes between the intervention and control groups of children living with HIV. Appendix 7. Age group stratified effectiveness of the intervention in improving oral and overall health outcomes between children living with HIV and without HIV. Appendix 8. Dentition type stratified longitudinal association between oral health outcomes and overall health outcomes changes in the intervention group. Appendix 9. Age group stratified longitudinal association between oral health outcomes and overall health outcomes changes in the intervention group. [file 12916_2023_2862_MOESM1_ESM.docx]

**Appendix 1**

**Training Schedule (example)**

| Session 1: Learn about tooth decay | | |
| --- | --- | --- |
| 1-1 | Quiz | Oral health quiz |
| 1-2 | Tooth decay | What is tooth decay? |
|  |  | Causes and prevention of tooth decay |
|  |  | Good and bad foods for teeth |
| Session 2: Practice of oral cleaning | | |
| 2-1 | Brushing | Appropriate brushing |
| 2-2 | Flossing | Appropriate flossing |
| Session 3: Know your oral health status | | |
| 3-1 | Check plaque status | Evaluate plaque status using plaque tester |
| 3-2 | Clean plaques | Clean plaques using toothpaste and tooth brushing |
| Session 4: Monitoring | | |
| 4-1 | Daily check | Instructions for performing the daily check |
| 4-2 | Schedule | Schedule of monitoring and the next appointment |
| 4-3 | Tools | Hand health diary, seals, tooth paste, tooth brush, dental floss |

**Appendix 2**

**Outcome measures**

| Outcomes | Measure | Person who collected | Methods of data collection | Timing of collection |
| --- | --- | --- | --- | --- |
| Overall health | Viral load | Research assistants | Registered medical records at the National Pediatric Hospital | Before the baseline survey period, and after the endline survey period |
| Overall health | Height for age | Research assistants | Measured height was converted to Z-score. | Baseline and endline survey |
| Overall health | Body mass index for age | Research assistants | Measured weight was converted to Z-score. | Baseline and endline survey |
| Overall health | Overall quality of life | Research assistants | Interview using a structured questionnaire | Baseline and endline survey |
| Oral health--oral hygiene | Decayed, missing, and filled teeth of primary and permanent teeth | Dentists of the National Pediatric Hospital | Oral examination. | Baseline and endline survey |
| Oral health--oral hygiene | Salivary pH | Dentists of the National Pediatric Hospital | Salivary test using kit (CAT21 Buf). | Baseline and endline survey |
| Oral health--oral hygiene | Salivary flow quantity | Dentists of the National Pediatric Hospital | Collection of saliva using chewing gum stimulation. | Baseline and endline survey |
| Oral health--oral hygiene | Debris index | Dentists of the National Pediatric Hospital | Oral examination. The value was calculated based on the amount of dental plaque on the dental surface. | Baseline and endline survey |
| Oral health--oral hygiene | Oral health related quality of life | Research assistants | Interview using a structured questionnaire | Baseline and endline survey |
| Oral health--oral health care behavior | Dental visits | Research assistants | Interview using a structured questionnaire | Baseline and endline survey |
| Oral health--oral health care behavior | Brushing frequency | Research assistants | Interview using a structured questionnaire | Baseline and endline survey |
| Oral health--oral health care behavior | Brushing duration | Research assistants | Interview using a structured questionnaire | Baseline and endline survey |
| Oral health--oral health care behavior | Caregivers’ oral care support | Research assistants | Interview using a structured questionnaire | Baseline and endline survey |

**Appendix 3. Participants’ characteristics at the baseline and endline surveys**

| Characteristics | |  | All participants | | |  | **Group A** Intervention group (HIV-positive) | | |  | **Group B** Control group (HIV-positive) | | |  | **Group C** Control group (HIV-negative) | | |
| --- | --- | --- | --- | --- | --- | --- | --- | --- | --- | --- | --- | --- | --- | --- | --- | --- | --- |
|  |  | Baseline (n=482) | | Endline (n= 350) | | Baseline (n=160) | | Endline  (n= 131) | | Baseline (n=168) | | Endline  (n=125) | | Baseline  (n=153) | | Endline  (n= 94) | |
| Child's sex, Female, n (%) | | 222 | 46.1 | 155 | 44.3 | 82 | 51.2 | 64 | 48.9 | 76 | 45.2 | 57 | 45.6 | 63 | 41.2 | 34 | 36.2 |
| Child's age, mean (SD) | | 10.8 | 2.9 | 13.6 | 2.9 | 10.7 | 3.1 | 13.8 | 3.1 | 10.9 | 2.9 | 14.1 | 2.8 | 10.7 | 2.9 | 12.59 | 2.6 |
| Dentition type | |  |  |  |  |  |  |  |  |  |  |  |  |  |  |  |  |
|  | Permanent teeth | 189 | 39.3 | 128 | 36.6 | 71 | 44.4 | 57 | 43.5 | 69 | 41.1 | 50 | 40.0 | 49 | 32.0 | 21 | 22.3 |
|  | Mixed dentition | 262 | 54.5 | 198 | 56.6 | 77 | 48.1 | 64 | 48.9 | 88 | 52.4 | 65 | 52.0 | 97 | 63.4 | 69 | 73.4 |
|  | Deciduous teeth | 30 | 6.2 | 24 | 6.9 | 12 | 7.5 | 10 | 7.6 | 11 | 6.5 | 10 | 8.0 | 7 | 4.6 | 4 | 4.3 |
| Child's overall health status | |  |  |  |  |  |  |  |  |  |  |  |  |  |  |  |  |
|  | Viral load detected *, n (%) | 104 | 31.7 | 39 | 15.3 | 50 | 31.3 | 21 | 16.2 | 54 | 32.1 | 18 | 14.4 | n/a | n/a | n/a | n/a |
|  | Height for age in z-score, mean (SD) | -2.0 | 1.5 | -1.6 | 1.1 | -2.0 | 1.41 | -1.8 | 1.0 | -1.8 | 1.39 | -1.7 | 1.0 | -2.2 | 1.5 | -1.1 | 1.1 |
|  | BMI for age in z-score, mean (SD) | -0.9 | 1.2 | -0.8 | 1.2 | -1.08 | 1.12 | -1.0 | 1.0 | -0.87 | 1.21 | -0.7 | 1.0 | -0.67 | 1.38 | -0.9 | 1.4 |
|  | Overall health-related quality of life, mean (SD) | 80.2 | 10.5 | 85.9 | 9.6 | 79.7 | 11.6 | 86.4 | 9.1 | 80.2 | 10.1 | 85.6 | 10.1 | 80.8 | 9.7 | 85.5 | 9.7 |
|  | Disease history*, n (%) | 261 | 79.6 | 46 | 18.0 | 126 | 78.8 | 30 | 22.9 | 135 | 80.4 | 16 | 12.8 | n/a | n/a | n/a | n/a |
|  | HIV-related health problem, mean (SD) | 401 | 83.4 | 277 | 79.1 | 131 | 81.9 | 98 | 74.8 | 142 | 84.5 | 97 | 77.6 | 128 | 83.7 | 82 | 87.2 |
| Child's oral hygiene status | |  |  |  |  |  |  |  |  |  |  |  |  |  |  |  |  |
|  | Oral Health related Quality of Life, mean (SD) | 12.0 | 7.3 | 8.6 | 6.7 | 11.9 | 7.6 | 8.1 | 6.0 | 11.8 | 7.2 | 7.9 | 6.0 | 12.4 | 7.0 | 10.1 | 8.1 |
|  | Decayed, Missing, Filled teeth, mean (SD) |  |  |  |  |  |  |  |  |  |  |  |  |  |  |  |  |
|  | Total teeth | 8.0 | 4.8 | 5.0 | 3.8 | 8.0 | 4.8 | 5.6 | 3.7 | 7.7 | 5.0 | 5.1 | 3.9 | 8.2 | 4.6 | 4.2 | 3.8 |
|  | Permanent teeth | 3.9 | 3.7 | 3.7 | 3.3 | 4.0 | 3.6 | 4.1 | 3.4 | 4.2 | 3.7 | 4.0 | 3.4 | 3.4 | 3.8 | 2.6 | 3.0 |
|  | Deciduous teeth | 7.1 | 4.7 | 4.8 | 4.1 | 7.6 | 4.6 | 4.9 | 3.6 | 6.5 | 5.1 | 4.7 | 4.4 | 7.2 | 4.4 | 4.7 | 4.5 |
|  | Salivary pH, mean (SD) | 6.1 | 0.5 | 6.2 | 0.5 | 6.0 | 0.6 | 6.1 | 0.5 | 6.0 | 0.6 | 6.1 | 0.5 | 6.2 | 0.4 | 6.2 | 0.4 |
|  | Salivary flow, mean (SD) | 2.7 | 1.3 | 3.4 | 1.3 | 2.9 | 1.3 | 3.4 | 1.4 | 2.8 | 1.3 | 3.3 | 1.2 | 2.2 | 1.3 | 3.6 | 1.3 |
|  | Debris index, mean (SD) | 2.2 | 0.7 | 0.9 | 0.7 | 2.1 | 0.7 | 0.9 | 0.7 | 2.1 | 0.7 | 0.8 | 0.7 | 2.4 | 0.7 | 1.1 | 0.7 |
| Under ART period, mean (SD)* | | 75.9 | 43.5 |  | - | 73.2 | 43.5 |  | - | 79.39 | 43.5 |  | - | n/a | n/a | n/a | n/a |
| Oral mucosal lesions* | |  |  |  |  |  |  |  |  |  |  |  |  |  |  |  |  |
|  | Have oral mucosal lesions | 53 | 16.2 | 17 | 6.6 | 26 | 16.3 | 11 | 8.5 | 27 | 16.1 | 6 | 4.8 | n/a | n/a | n/a | n/a |
|  | Abscess | 49 | 14.9 | 13 | 5.0 | 26 | 16.3 | 10 | 7.7 | 23 | 13.7 | 3 | 2.4 | n/a | n/a | n/a | n/a |
|  | Ulceration | 4 | 1.2 | 0 | 0 | 1 | 0.6 | 0 | 0 | 3 | 1.8 | 0 | 0 | n/a | n/a | n/a | n/a |
|  | Acute necrotizing ulcerative gingivitis | 2 | 0.6 | 1 | 0.3 | 0 | 0 | 1 | 0.8 | 1 | 0 | 0 | 0 | n/a | n/a | n/a | n/a |
|  | Candidasis | 0 | 0 | 3 | 1.1 | 0 | 0 | 0 | 0 | 0 | 0 | 3 | 2.4 | n/a | n/a | n/a | n/a |
| Child's oral care behavior | |  |  |  |  |  |  |  |  |  |  |  |  |  |  |  |  |
|  | Visited a dentist in the past 12 months for pain or trouble with teeth, n (%) | 99 | 22.0 | 53 | 15.2 | 44 | 29.7 | 15 | 11.5 | 31 | 19.4 | 16 | 12.9 | 24 | 16.8 | 22 | 23.7 |
|  | Frequency of tooth brushing per day, n (%) |  |  |  |  |  |  |  |  |  |  |  |  |  |  |  |  |
|  | Three times or more | 115 | 25.4 | 129 | 37.1 | 41 | 27.5 | 64 | 48.9 | 40 | 25.0 | 38 | 30.6 | 34 | 23.8 | 27 | 29.0 |
|  | Twice a day | 133 | 29.4 | 180 | 51.7 | 47 | 31.5 | 59 | 45.0 | 48 | 30.0 | 66 | 53.2 | 38 | 26.6 | 55 | 59.1 |
|  | Once a day or less | 93 | 20.5 | 39 | 11.2 | 34 | 22.8 | 8 | 6.1 | 38 | 23.8 | 20 | 16.1 | 21 | 14.7 | 11 | 11.9 |
|  | Never clean teeth | 111 | 24.6 | 0 | 0 | 27 | 18.1 | 0 | 0 | 34 | 21.3 | 0 | 0 | 50 | 35.0 | 0 | 0 |
|  | Duration of brushing, n (%) |  |  |  |  |  |  |  |  |  |  |  |  |  |  |  |  |
|  | ≤1 min | 118 | 27.8 | 65 | 18.8 | 49 | 35.5 | 13 | 10.1 | 39 | 25.8 | 23 | 18.5 | 30 | 22.2 | 29 | 31.2 |
|  | 2 mins | 99 | 23.3 | 79 | 22.8 | 27 | 19.6 | 24 | 18.6 | 35 | 23.2 | 34 | 27.4 | 37 | 27.4 | 21 | 22.6 |
|  | ≥ 3mins | 207 | 48.8 | 202 | 58.4 | 62 | 44.9 | 92 | 71.3 | 77 | 51.0 | 67 | 54 | 68 | 50.4 | 43 | 46.2 |
| Caregiver's help for the child's teeth brushing, n (%) | |  |  |  |  |  |  |  |  |  |  |  |  |  |  |  |  |
|  | Caregiver brush child's teeth or watch/advise while child is brushing | 27 | 6.1 | 21 | 15.8 | 13 | 9.1 | 25 | 19.1 | 11 | 6.9 | 13 | 10.5 | 3 | 2.2 | 17 | 18.3 |
|  | Caregiver has never cared about the child's teeth brushing | 412 | 93.8 | 293 | 84.2 | 130 | 90.9 | 106 | 80.9 | 146 | 93.0 | 111 | 89.5 | 136 | 97.8 | 76 | 81.7 |
| Family wealth index, n (%) | |  |  |  |  |  |  |  |  |  |  |  |  |  |  |  |  |
|  | Lowest | 95 | 19.8 |  |  | 28 | 17.5 |  |  | 52 | 31.0 |  |  | 15 | 9.9 |  |  |
|  | Highest | 93 | 19.4 |  |  | 41 | 25.6 |  |  | 21 | 12.5 |  |  | 31 | 20.5 |  |  |
| Parents living status, n (%) | |  |  |  |  |  |  |  |  |  |  |  |  |  |  |  |  |
|  | Both parents alive |  |  | 226 | 65.0 |  |  | 65 | 50.0 |  |  | 74 | 60.2 |  |  | 86 | 92.5 |
|  | Only father alive |  |  | 39 | 11.1 |  |  | 21 | 16.2 |  |  | 18 | 14.6 |  |  | 0 | 0 |
|  | Only mother alive |  |  | 40 | 11.4 |  |  | 17 | 13.1 |  |  | 17 | 13.8 |  |  | 6 | 6.5 |
|  | Both parents not alive |  |  | 42 | 12.0 |  |  | 27 | 20.8 |  |  | 14 | 11.4 |  |  | 1 | 1.1 |

*Group C was excluded from the total participants number

**Appendix 4. Followed-up and lost-to-follow up participants**

| Characteristics | | All participants | | | | | | | | | | **Group A** Intervention group (HIV-positive) | | | | | | | | | | | | **Group B** Control group (HIV-positive) | | | | | | | | | | | | **Group C** Control group (HIV-negative) | | | | | | | | | | | |
| --- | --- | --- | --- | --- | --- | --- | --- | --- | --- | --- | --- | --- | --- | --- | --- | --- | --- | --- | --- | --- | --- | --- | --- | --- | --- | --- | --- | --- | --- | --- | --- | --- | --- | --- | --- | --- | --- | --- | --- | --- | --- | --- | --- | --- | --- | --- | --- |
|  |  | Lost to follow-up  (n=132) | | Followed (n= 350) | | | | p-value | | | | Lost to follow-up  (n=29) | | | Followed  (n= 131) | | | | | p-value | | | | Lost to follow-up  (n=43) | | | | Followed (n=125) | | | | p-value | | | | Lost to follow-up  (n=60) | | | | Endline  (n= 94) | | | | | p-value | | |
|  |  |  |  | |  | |  | | |  | |  | |  | | |  | |  | | |  | |  | |  | | |  | |  | | |  | |  | |  | | |  | |  | | | |  |
| Child's sex, Female, n (%) | | 66 | 50.4 | | 155 | | 44.3 | | | 0.20 | | 18 | | 62.1 | | | 64 | | 48.9 | | | 0.20 | | 19 | | 44.2 | | | 57 | | 45.6 | | | 0.87 | | 29 | | 49.2 | | | 34 | | 36.2 | | | | 0.09 |
| Child's age, mean (SD) | | 11.2 | 2.9 | | 10.6 | | 3.0 | | | 0.07 | | 10.6 | | 3.3 | | | 10.8 | | 3.1 | | | 0.78 | | 11.4 | | 3.0 | | | 10.8 | | 2.8 | | | 0.27 | | 11.3 | | 2.8 | | | 10.2 | | 3.0 | | | | 0.02 |
| Dentition type | |  |  | |  | |  | | | 0.10 | |  | |  | | |  | |  | | | 0.90 | |  | |  | | |  | |  | | | 0.42 | |  | |  | | |  | |  | | | | 0.01 |
|  | Permanent teeth | 61 | 46.2 | | 128 | | 36.6 | | |  | | 14 | | 48.3 | | | 57 | | 43.5 | | |  | | 19 | | 44.2 | | | 50 | | 40.0 | | |  | | 28 | | 46.7 | | | 21 | | 22.3 | | | |  |
|  | Mixed dentition | 66 | 50.0 | | 197 | | 56.3 | | |  | | 13 | | 44.8 | | | 64 | | 48.9 | | |  | | 23 | | 53.5 | | | 65 | | 52.0 | | |  | | 30 | | 50 | | | 68 | | 72.3 | | | |  |
|  | Deciduous teeth | 5 | 3.8 | | 25 | | 7.1 | | |  | | 2 | | 6.9 | | | 10 | | 7.6 | | |  | | 1 | | 2.3 | | | 10 | | 8.0 | | |  | | 2 | | 3.3 | | | 5 | | 5.3 | | | |  |
| Child's overall health status | |  |  | |  | |  | | |  | |  | |  | | |  | |  | | |  | |  | |  | | |  | |  | | |  | |  | |  | | |  | |  | | | |  |
|  | Viral load detected *, n (%) | 25 | 34.7 | | 79 | | 30.9 | | | 0.53 | | 11 | | 37.9 | | | 39 | | 29.8 | | | 0.39 | | 14 | | 32.6 | | | 40 | | 32 | | | 0.95 | | n/a | | n/a | | | n/a | | n/a | | | | n/a |
|  | Height for age in z-score, mean (SD) | -1.9 | 1.4 | | -2.0 | | 1.5 | | | 0.38 | | -1.6 | | 1.1 | | | -2.0 | | 1.5 | | | 0.15 | | -1.9 | | 1.3 | | | -1.8 | | 1.4 | | | 0.61 | | -1.9 | | 1.6 | | | -2.4 | | 1.5 | | | | 0.18 |
|  | BMI for age in z-score, mean (SD) | -0.8 | 1.2 | | -0.9 | | 1.3 | | | 0.90 | | -0.9 | | 1.1 | | | -1.1 | | 1.1 | | | 0.83 | | -0.84 | | 0.9 | | | -0.9 | | 1.3 | | | 0.71 | | -0.8 | | 1.4 | | | -0.6 | | 1.4 | | | | 0.55 |
|  | Overall health-related quality of life, mean (SD) | 80.9 | 9.9 | | 80.0 | | 10.7 | | | 0.42 | | 78.7 | | 12.8 | | | 79.9 | | 11.3 | | | 0.74 | | 81.5 | | 9.0 | | | 79.7 | | 10.5 | | | 0.43 | | 81.7 | | 8.8 | | | 80.3 | | 10.3 | | | | 0.47 |
| Child's oral hygiene status | |  |  | |  | |  | | |  | |  | |  | | |  | |  | | |  | |  | |  | | |  | |  | | |  | |  | |  | | |  | |  | | | |  |
|  | Oral Health related Quality of Life, mean (SD) | 11.7 | 7.2 | | 12.1 | | 7.3 | | | 0.59 | | 13.2 | | 8.3 | | | 11.6 | | 7.4 | | | 0.45 | | 11.2 | | 6.6 | | | 11.9 | | 7.4 | | | 0.66 | | 11.4 | | 7.1 | | | 12.7 | | 7.1 | | | | 0.21 |
|  | Decayed, Missing, Filled teeth, mean (SD) |  |  | |  | |  | | |  | |  | |  | | |  | |  | | |  | |  | |  | | |  | |  | | |  | |  | |  | | |  | |  | | | |  |
|  | Total teeth (n=482) | 8.0 | 5.1 | | 7.9 | | 4.7 | | | 0.75 | | 8.7 | | 5.2 | | | 7.8 | | 4.8 | | | 0.39 | | 8.3 | | 5.5 | | | 7.51 | | 4.9 | | | 0.41 | | 7.6 | | 5.0 | | | 8.6 | | 4.4 | | | | 0.22 |
|  | Permanent teeth | 4.0 | 4.0 | | 3.8 | | 3.6 | | | 0.97 | | 4.4 | | 4.0 | | | 3.9 | | 3.5 | | | 0.82 | | 3.9 | | 3.6 | | | 4.3 | | 3.8 | | | 0.54 | | 3.9 | | 4.2 | | | 3.0 | | 3.4 | | | | 0.36 |
|  | Deciduous teeth | 7.8 | 5.0 | | 6.9 | | 4.7 | | | 0.16 | | 8.9 | | 4.2 | | | 7.3 | | 4.7 | | | 0.25 | | 8.1 | | 5.9 | | | 5.93 | | 4.8 | | | 0.12 | | 7.2 | | 4.7 | | | 7.4 | | 4.4 | | | | 0.80 |
|  | Salivary pH, mean (SD) | 6.0 | 0.5 | | 6.1 | | 0.5 | | | 0.10 | | 5.9 | | 0.6 | | | 6.1 | | 0.6 | | | 0.11 | | 5.9 | | 0.6 | | | 6.0 | | 0.6 | | | 0.24 | | 6.2 | | 0.4 | | | 6.2 | | 0.4 | | | | 0.34 |
|  | Salivary flow, mean (SD) | 2.5 | 1.2 | | 2.7 | | 1.4 | | | 0.15 | | 2.4 | | 1.2 | | | 3.0 | | 1.3 | | | 0.02 | | 2.9 | | 1.3 | | | 2.8 | | 1.3 | | | 0.76 | | 2.3 | | 1.2 | | | 2.2 | | 1.3 | | | | 0.33 |
|  | Debris index, mean (SD) | 2.2 | 0.7 | | 2.2 | | 0.7 | | | 0.60 | | 2.1 | | 0.7 | | | 2.2 | | 0.7 | | | 0.33 | | 2.2 | | 0.8 | | | 2.1 | | 0.7 | | | 0.56 | | 2.3 | | 0.7 | | | 2.4 | | 0.6 | | | | 0.84 |
| Child's oral care behavior | |  |  | |  | |  | | |  | |  | |  | | |  | |  | | |  | |  | |  | | |  | |  | | |  | |  | |  | | |  | |  | | | |  |
|  | Visited a dentist in the past 12 months for pain or trouble with teeth, n (%) | 23 | 18.1 | | 76 | | 23.5 | | | 0.22 | | 7 | | 26.9 | | | 37 | | 30.3 | | | 0.73 | | 8 | | 18.6 | | | 23 | | 19.7 | | | 0.88 | | 8 | | 13.8 | | | 16 | | 18.8 | | | | 0.43 |
|  | Frequency of tooth brushing per day, n (%) |  |  | |  | |  | | |  | |  | |  | | |  | |  | | |  | |  | |  | | |  | |  | | |  | |  | |  | | |  | |  | | | |  |
|  | Three times or more | 29 | 22.7 | | 86 | | 26.5 | | | 0.32 | | 7 | | 25.9 | | | 34 | | 27.9 | | | 0.03 | | 9 | | 20.9 | | | 31 | | 26.5 | | | 0.07 | | 13 | | 22.4 | | | 21 | | 24.7 | | | | 0.77 |
|  | Twice a day | 31 | 24.2 | | 102 | | 31.5 | | |  | | 7 | | 25.9 | | | 40 | | 32.8 | | |  | | 9 | | 20.9 | | | 39 | | 33.3 | | |  | | 15 | | 25.9 | | | 23 | | 27.1 | | | |  |
|  | Once a day or less | 32 | 24.9 | | 61 | | 18.9 | | |  | | 8 | | 29.6 | | | 26 | | 47.3 | | |  | | 16 | | 37.2 | | | 22 | | 18.8 | | |  | | 8 | | 13.7 | | | 13 | | 15.3 | | | |  |
|  | Never clean teeth | 36 | 28.1 | | 75 | | 23.1 | | |  | | 5 | | 18.5 | | | 22 | | 18.0 | | |  | | 9 | | 20.9 | | | 25 | | 21.4 | | |  | | 22 | | 37.9 | | | 28 | | 32.9 | | | |  |
|  | Duration of brushing, n (%) |  |  | |  | |  | | |  | |  | |  | | |  | |  | | |  | |  | |  | | |  | |  | | |  | |  | |  | | |  | |  | | | |  |
|  | ≤1 min | 27 | 22.5 | | 91 | | 29.9 | | | 0.25 | | 7 | | 26.9 | | | 42 | | 37.5 | | | 0.54 | | 11 | | 27.5 | | | 28 | | 29.2 | | | 0.76 | | 9 | | 16.7 | | | 21 | | 26.0 | | | | 0.25 |
|  | 2 mins | 33 | 27.5 | | 66 | | 21.7 | | |  | | 7 | | 26.9 | | | 20 | | 17.9 | | |  | | 7 | | 17.5 | | | 28 | | 25.2 | | |  | | 19 | | 35.2 | | | 18 | | 22.2 | | | |  |
|  | ≥ 3mins | 60 | 50.0 | | 147 | | 48.4 | | |  | | 12 | | 46.2 | | | 50 | | 44.6 | | |  | | 22 | | 55.0 | | | 55 | | 49.5 | | |  | | 26 | | 48.1 | | | 42 | | 51.9 | | | |  |
| Caregiver's help for the child's teeth brushing, n (%) | |  |  | |  | |  | | |  | |  | |  | | |  | |  | | |  | |  | |  | | |  | |  | | |  | |  | |  | | |  | |  | | | |  |
|  | Caregiver brush child's teeth or watch/advise while child is brushing | 6 | 4.8 | | 21 | | 6.7 | | | 0.53 | | 3 | | 11.5 | | | 10 | | 8.5 | | | 0.92 | | 2 | | 4.9 | | | 9 | | 7.8 | | | 0.30 | | 1 | | 1.8 | | | 2 | | 2.4 | | | | 0.79 |
|  | Caregiver has never cared about the child's teeth brushing | 118 | 95.2 | | 294 | | 93.3 | | |  | | 23 | | 88.5 | | | 107 | | 91.5 | | |  | | 39 | | 95.1 | | | 107 | | 92.2 | | |  | | 56 | | 98.2 | | | 80 | | 97.6 | | | |  |
| Family wealth index, n (%) | |  |  | |  | |  | | |  | |  | |  | | |  | |  | | |  | |  | |  | | |  | |  | | |  | |  | |  | | |  | |  | | | |  |
|  | Lowest | 27 | 20.6 | | 68 | | 19.5 | | | 0.33 | | 5 | | 17.2 | | | 23 | | 17.6 | | | 0.09 | | 16 | | 37.2 | | | 36 | | 28.8 | | | 0.65 | | 6 | | 10.2 | | | 9 | | 9.7 | | | | 0.98 |
|  | Highest | 18 | 13.7 | | 75 | | 21.5 | | |  | | 3 | | 10.3 | | | 38 | | 29.0 | | |  | | 3 | | 7.0 | | | 18 | | 14.4 | | |  | | 12 | | 20.3 | | | 19 | | 20.4 | | | |  |
|  | *Group C was excluded from the total participants number |  | | | |  |  | |  | |  | |  | | |  | |  | | |  | |  | |  | |  | | |  | | |  | |  | |  | |  | | |  | |  | |  | |

**Appendix 5. Differences of baseline and endline oral hygiene status in each participant group**

| **Group A** |  |  |  |  |  |  |  |  |
| --- | --- | --- | --- | --- | --- | --- | --- | --- |
| Oral hygiene indicators | Baseline | Endline | Paired-test P-value | Generalized Estimating Equations | | | |  |
|  |  |  |  | B | 95% CI | | P-value |  |
| VL detected (%)* | 30.4 | 15.3 | **0.001** | -0.664 | -1.232 | -0.095 | **0.022** |  |
| Salivary pH** (mean) | 6.1 | 6.1 | 0.221 | 0.002 | -0.007 | 0.012 | 0.627 |  |
| Salivary flow** (ml/min) | 3.03 | 3.44 | **<0.001** | 0.034 | -0.005 | 0.074 | 0.086 |  |
| DMFT ≤5 (%)* | 55.5 | 58.6 | **<0.001** | 0.336 | -0.103 | 0.774 | 0.134 |  |
| Debris index** | 2.17 | 0.89 | **<0.001** | -0.198 | -0.229 | -0.167 | **<0.001** |  |
| OHQOL** | 11.76 | 8.14 | **<0.001** | -0.138 | -0.219 | -0.057 | **0.001** |  |
|  |  |  |  |  |  |  |  |  |
| **Group B** |  |  |  |  |  |  |  |  |
| Oral hygiene indicators | Baseline | Endline | Paired-test P-value | Generalized Estimating Equations | | | |  |
|  |  |  |  | B | 95% CI | | P-value |  |
| VL detected (%)* | 32.0 | 14.4 | **<0.001** | -0.684 | -1.343 | -0.024 | **0.042** |  |
| Salivary pH** (mean) | 6.0 | 6.1 | 0.153 | -0.002 | -0.013 | 0.009 | 0.695 |  |
| Salivary flow** (ml/min) | 2.79 | 3.25 | **0.002** | 0.032 | -0.023 | 0.086 | 0.252 |  |
| DMFT ≤5 (%)* | 38.4 | 58.1 | **0.001** | 0.49 | -0.039 | 1.019 | 0.069 |  |
| Debris index** | 2.12 | 0.785 | **<0.001** | -0.209 | -0.244 | -0.175 | **<0.001** |  |
| OHQOL** | 11.91 | 7.92 | **<0.001** | -0.147 | -0.229 | -0.066 | **<0.001** |  |
|  |  |  |  |  |  |  |  |  |
| **Group C** |  |  |  |  |  |  |  |  |
| Oral hygiene indicators | Baseline | Endline | Paired-test P-value | Generalized Estimating Equations | | | |  |
|  |  |  |  | B | 95% CI | | P-value |  |
| Salivary pH** (mean) | 6.2 | 6.2 | 0.781 | -0.001 | -0.008 | 0.006 | 0.814 |  |
| Salivary flow** (ml/min) | 2.15 | 3.57 | **<0.001** | 0.244 | 0.179 | 0.309 | **<0.001** |  |
| DMFT ≤5 (%)* | 29.5 | 63.8 | **<0.001** | 0.987 | 0.372 | 1.602 | **0.002** |  |
| Debris index** | 2.36 | 1.057 | **<0.001** | -0.277 | -0.356 | -0.198 | **<0.001** |  |
| OHQOL** | 12.7 | 10.03 | **<0.001** | -0.141 | -0.239 | -0.043 | **0.005** |  |
|  |  |  |  |  |  |  |  |  |
| Participants who had both baseline and endline data were analyzed. | | |  |  |  |  |  |  |
| Paired-test: McNemar* and Wilcoxon signed-rank test** | |  |  |  |  |  |  |  |
| Generalized Estimating Equations: adjusted with infant's age, infant's sex, and survey type (baseline and endline) | | | | | | |  |  |

**Appendix 6. Age group stratified effect of the intervention on oral and overall health outcomes between the intervention and control groups of children living with HIV**

| Variables | Intervention x Time | | | | | | | | | | | | | | | | | | | | | | | | | | | | | | | | | | | | | |
| --- | --- | --- | --- | --- | --- | --- | --- | --- | --- | --- | --- | --- | --- | --- | --- | --- | --- | --- | --- | --- | --- | --- | --- | --- | --- | --- | --- | --- | --- | --- | --- | --- | --- | --- | --- | --- | --- | --- |
|  | All age groups | | | | | | | | | 6-8 years old at baseline | | | | | | | | | | 9-12 years old at baseline | | | | | | | | | 13-15 years old at baseline | | | | | | | | | |
|  | Estimate | | | 95% CI | | | P Value | | | | Estimate | | | 95% CI | | | P Value | | | Estimate | | | 95% CI | | | P Value | | | | Estimate | | | 95% CI | | | P Value | | |
| Viral load detected (n, %) |  |  | | |  | | | |  | |  |  | | | |  | | |  |  |  | | |  | | | |  | |  |  | | | |  | | |  |
| Baseline |  |  | | |  | | | |  | |  |  | | | |  | | |  |  |  | | |  | | | |  | |  |  | | | |  | | |  |
| Endline | 1.25 | 0.58 | | | 2.69 | | | | 0.56 | | 0.35 | 0.06 | | | | 2.02 | | | 0.23 | 1.29 | 0.37 | | | 4.57 | | | | 0.69 | | 3.65 | 0.80 | | | | 16.64 | | | 0.09 |
| Height for age (Mean, SD) |  |  | | |  | | | |  | |  |  | | | |  | | |  |  |  | | |  | | | |  | |  |  | | | |  | | |  |
| Baseline |  |  | | |  | | | |  | |  |  | | | |  | | |  |  |  | | |  | | | |  | |  |  | | | |  | | |  |
| Endline | 0.01 | -0.01 | | | 0.03 | | | | 0.32 | | 0.04 | -0.03 | | | | 0.10 | | | 0.27 | 0.01 | -0.01 | | | 0.03 | | | | 0.25 | | -0.02 | -0.04 | | | | 0.01 | | | 0.22 |
| Body-mass-index for age (Mean, SD) |  |  | | |  | | | |  | |  |  | | | |  | | |  |  |  | | |  | | | |  | |  |  | | | |  | | |  |
| Baseline |  |  | | |  | | | |  | |  |  | | | |  | | |  |  |  | | |  | | | |  | |  |  | | | |  | | |  |
| Endline | -0.02 | -0.05 | | | 0.01 | | | | 0.15 | | -0.02 | -0.11 | | | | 0.07 | | | 0.66 | -0.01 | -0.05 | | | 0.02 | | | | 0.46 | | -0.03 | -0.07 | | | | <0.01 | | | 0.07 |
| Overall QOL (Mean, SD) |  |  | | |  | | | |  | |  |  | | | |  | | |  |  |  | | |  | | | |  | |  |  | | | |  | | |  |
| Baseline |  |  | | |  | | | |  | |  |  | | | |  | | |  |  |  | | |  | | | |  | |  |  | | | |  | | |  |
| Endline | <0.01 | -0.02 | | | 0.02 | | | | 0.69 | | -0.02 | -0.07 | | | | 0.03 | | | 0.50 | 0.02 | -0.01 | | | 0.05 | | | | 0.27 | | <0.01 | -0.03 | | | | 0.03 | | | 0.99 |
| OHQOL (Mean, SD) |  |  | | |  | | | |  | |  |  | | | |  | | |  |  |  | | |  | | | |  | |  |  | | | |  | | |  |
| Baseline |  |  | | |  | | | |  | |  |  | | | |  | | |  |  |  | | |  | | | |  | |  |  | | | |  | | |  |
| Endline | 0.01 | -0.04 | | | 0.05 | | | | 0.77 | | 0.12 | -0.02 | | | | 0.25 | | | 0.09 | -0.04 | -0.10 | | | 0.03 | | | | 0.23 | | 0.02 | -0.05 | | | | 0.08 | | | 0.61 |
| Salivary pH (Mean, SD) |  |  | | |  | | | |  | |  |  | | | |  | | |  |  |  | | |  | | | |  | |  |  | | | |  | | |  |
| Baseline |  |  | | |  | | | |  | |  |  | | | |  | | |  |  |  | | |  | | | |  | |  |  | | | |  | | |  |
| Endline | <-0.01 | -0.01 | | | 0.01 | | | | 0.73 | | 0.01 | -0.02 | | | | 0.05 | | | 0.40 | <-0.01 | -0.02 | | | 0.02 | | | | 0.73 | | -0.01 | -0.02 | | | | 0.01 | | | 0.59 |
| Salivary flow (Mean, SD) |  |  | | |  | | | |  | |  |  | | | |  | | |  |  |  | | |  | | | |  | |  |  | | | |  | | |  |
| Baseline |  |  | | |  | | | |  | |  |  | | | |  | | |  |  |  | | |  | | | |  | |  |  | | | |  | | |  |
| Endline | -0.03 | -0.09 | | | 0.03 | | | | 0.38 | | -0.09 | -0.25 | | | | 0.08 | | | 0.32 | -0.03 | -0.12 | | | 0.05 | | | | 0.44 | | 0.04 | -0.07 | | | | 0.14 | | | 0.50 |
| DMFT/dmft (total teeth) (Mean, SD) |  |  | | |  | | | |  | |  |  | | | |  | | |  |  |  | | |  | | | |  | |  |  | | | |  | | |  |
| Baseline |  |  | | |  | | | |  | |  |  | | | |  | | |  |  |  | | |  | | | |  | |  |  | | | |  | | |  |
| Endline | 0.04 | -0.03 | | | 0.11 | | | | 0.27 | | 0.11 | -0.06 | | | | 0.28 | | | 0.21 | 0.06 | -0.06 | | | 0.18 | | | | 0.32 | | 0.01 | -0.10 | | | | 0.11 | | | 0.92 |
| DMFT (permanent teeth) (Mean, SD) |  |  | | |  | | | |  | |  |  | | | |  | | |  |  |  | | |  | | | |  | |  |  | | | |  | | |  |
| Baseline |  |  | | |  | | | |  | |  |  | | | |  | | |  |  |  | | |  | | | |  | |  |  | | | |  | | |  |
| Endline | 0.05 | -0.02 | | | 0.12 | | | | 0.14 | | 0.04 | -0.20 | | | | 0.28 | | | 0.52 | 0.09 | -0.01 | | | 0.19 | | | | 0.07 | | -0.04 | -0.14 | | | | 0.07 | | | 0.50 |
| dmft (deciduous teeth) (Mean, SD) |  |  | | |  | | | |  | |  |  | | | |  | | |  |  |  | | |  | | | |  | |  |  | | | |  | | |  |
| Baseline |  |  | | |  | | | |  | |  |  | | | |  | | |  |  |  | | |  | | | |  | |  |  | | | |  | | |  |
| Endline | -0.03 | -0.17 | | | 0.12 | | | | 0.70 | | 0.07 | -0.12 | | | | 0.267 | | | 0.44 | 0.04 | -0.13 | | | 0.22 | | | | 0.61 | | n/a | n/a | | | | n/a | | | n/a |
| Debris index (Mean, SD) |  |  | | |  | | | |  | |  |  | | | |  | | |  |  |  | | |  | | | |  | |  |  | | | |  | | |  |
| Baseline |  |  | | |  | | | |  | |  |  | | | |  | | |  |  |  | | |  | | | |  | |  |  | | | |  | | |  |
| Endline | 0.01 | -0.03 | | | 0.05 | | | | 0.63 | | -0.03 | -0.13 | | | | -0.07 | | | 0.58 | 0.03 | -0.02 | | | 0.09 | | | | 0.22 | | <0.01 | -0.07 | | | | 0.07 | | | 0.96 |
| Visited a dentist for pain or trouble with teeth (n, %) |  |  | | |  | | | |  | |  |  | | | |  | | |  |  |  | | |  | | | |  | |  |  | | | |  | | |  |
| Baseline |  |  | | |  | | | |  | |  |  | | | |  | | |  |  |  | | |  | | | |  | |  |  | | | |  | | |  |
| Endline | 0.48 | 0.21 | | | 1.12 | | | | 0.09 | | 0.91 | 0.11 | | | | 7.83 | | | 0.93 | 0.86 | 0.27 | | | 2.79 | | | | 0.80 | | **0.08** | **0.01** | | | | **0.55** | | | **0.01** |
| Brush three times and more per day (n, %) |  |  | | |  | | | |  | |  |  | | | |  | | |  |  |  | | |  | | | |  | |  |  | | | |  | | |  |
| Baseline |  |  | | |  | | | |  | |  |  | | | |  | | |  |  |  | | |  | | | |  | |  |  | | | |  | | |  |
| Endline | **2.06** | **1.03** | | | **4.12** | | | | **0.03** | | 3.37 | 0.22 | | | | 51.92 | | | 0.37 | 1.23 | 0.48 | | | 3.18 | | | | 0.66 | | 2.58 | 0.84 | | | | 7.91 | | | 0.10 |
| Brush for three minutes or more (n, %) |  |  | | |  | | | |  | |  |  | | | |  | | |  |  |  | | |  | | | |  | |  |  | | | |  | | |  |
| Baseline |  |  | | |  | | | |  | |  |  | | | |  | | |  |  |  | | |  | | | |  | |  |  | | | |  | | |  |
| Endline | **2.69** | **1.37** | | | **5.31** | | | | **<0.01** | | 4.29 | 0.63 | | | | 29.20 | | | 0.13 | 1.78 | 0.69 | | | 4.60 | | | | 0.23 | | **5.20** | **1.44** | | | | **18.82** | | | **0.01** |
| Caregiver never care child's toothbrushing (n, %) |  |  | | |  | | | |  | |  |  | | | |  | | |  |  |  | | |  | | | |  | |  |  | | | |  | | |  |
| Baseline |  |  | | |  | | | |  | |  |  | | | |  | | |  |  |  | | |  | | | |  | |  |  | | | |  | | |  |
| Endline | 0.51 | 0.13 | | | 1.93 | | | | 0.32 | | 0.44 | 0.04 | | | | 4.85 | | | 0.49 | 0.30 | 0.04 | | | 2.26 | | | | 0.24 | | n/a | n/a | | | | n/a | | | n/a |
|  |  | |  | | |  | |  | |  | | |  | |  | | |  | |  | |  | | |  | |  | |  | | |  | |  | | |  | |

Adjusted with age, sex, time (baseline or endline), interaction of each variable and time for all participants' analyses. Age variable was not adjusted for the age stratified model.

OHQOL: Oral health related quality of life, DMFT: Decayed, missing filled teeth

**Appendix 7. Age group stratified effectiveness of the intervention in improving oral and overall health outcomes between children living with HIV and without HIV**

| Variables |  | Intervention x Time | | | | | | | | | | | | | | | | | |
| --- | --- | --- | --- | --- | --- | --- | --- | --- | --- | --- | --- | --- | --- | --- | --- | --- | --- | --- | --- |
|  |  | All age groups | | | | 6-8 years old at baseline | | | | 9-12 years old at baseline | | | | 13-15 years old at baseline | | | | | |
|  |  | Estimate | 95% CI | | P Value | Estimate | 95% CI | | P Value | Estimate | 95% CI | | P Value | Estimate | 95% CI | | P Value | | |
| Height for age (Mean, SD) |  |  |  |  |  |  |  |  |  |  |  |  |  |  |  |  |  | | |
| Baseline |  |  |  |  |  |  |  |  |  |  |  |  |  |  |  |  |  | | |
| Endline |  | **-0.06** | **-0.08** | **-0.04** | **<0.01** | -0.05 | -0.12 | 0.03 | 0.21 | **-0.07** | **-0.10** | **-0.04** | **<0.01** | **-0.07** | **-0.10** | **-0.03** | **<0.01** | | |
| Body-mass-index for age (Mean, SD) |  |  |  |  |  |  |  |  |  |  |  |  |  |  |  |  |  | | |
| Baseline |  |  |  |  |  |  |  |  |  |  |  |  |  |  |  |  |  | | |
| Endline |  | 0.03 | <0.01 | 0.07 | 0.05 | <0.01 | -0.09 | 0.09 | 1.00 | 0.06 | -0.02 | 0.13 | 0.16 | 0.03 | -0.02 | 0.07 | 0.29 | | |
| Overall QOL (Mean, SD) |  |  |  |  |  |  |  |  |  |  |  |  |  |  |  |  |  | | |
| Baseline |  |  |  |  |  |  |  |  |  |  |  |  |  |  |  |  |  | | |
| Endline |  | 0.01 | -0.01 | 0.03 | 0.51 | -0.01 | -0.07 | 0.04 | 0.60 | 0.01 | -0.02 | 0.03 | 0.70 | <0.01 | -0.03 | 0.04 | 0.88 | | |
| OHQOL (Mean, SD) |  |  |  |  |  |  |  |  |  |  |  |  |  |  |  |  |  | | |
| Baseline |  |  |  |  |  |  |  |  |  |  |  |  |  |  |  |  |  | | |
| Endline |  | <0.01 | -0.05 | 0.06 | 0.92 | 0.11 | -0.01 | 0.22 | 0.08 | -0.02 | -0.10 | 0.07 | 0.69 | -0.01 | -0.09 | 0.07 | 0.78 | | |
| Salivary pH (Mean, SD) |  |  |  |  |  |  |  |  |  |  |  |  |  |  |  |  |  | | |
| Baseline |  |  |  |  |  |  |  |  |  |  |  |  |  |  |  |  |  | | |
| Endline |  | <0.01 | -0.01 | 0.02 | 0.51 | <0.01 | -0.02 | 0.03 | 0.83 | 0.01 | -0.01 | 0.03 | 0.16 | <0.01 | -0.03 | 0.02 | 0.67 | | |
| Salivary flow (Mean, SD) |  |  |  |  |  |  |  |  |  |  |  |  |  |  |  |  |  | | |
| Baseline |  |  |  |  |  |  |  |  |  |  |  |  |  |  |  |  |  | | |
| Endline |  | **-0.20** | **-0.27** | **-0.13** | **<0.01** | -0.16 | -0.36 | 0.05 | 0.13 | **-0.17** | **-0.27** | **-0.06** | **<0.01** | **-0.22** | **-0.33** | **-0.10** | **<0.01** | | |
| DMFT/dmft (total teeth) (Mean, SD) |  |  |  |  |  |  |  |  |  |  |  |  |  |  |  |  |  | | |
| Baseline |  |  |  |  |  |  |  |  |  |  |  |  |  |  |  |  |  | | |
| Endline |  | **0.23** | **0.16** | **0.31** | **<0.01** | **0.24** | **0.08** | **0.40** | **<0.01** | **0.26** | **-0.14** | **0.38** | **<0.01** | **0.16** | **0.04** | **0.28** | **0.01** | | |
| DMFT (permanent teeth) (Mean, SD) |  |  |  |  |  |  |  |  |  |  |  |  |  |  |  |  |  | | |
| Baseline |  |  |  |  |  |  |  |  |  |  |  |  |  |  |  |  |  | | |
| Endline |  | 0.08 | <0.01 | 0.16 | 0.05 | 0.17 | -0.05 | 0.39 | 0.13 | 0.11 | -0.02 | 0.23 | 0.09 | 0.11 | <0.01 | 0.22 | 0.05 | | |
| dmft (deciduous teeth) (Mean, SD) |  |  |  |  |  |  |  |  |  |  |  |  |  |  |  |  |  | | |
| Baseline |  |  |  |  |  |  |  |  |  |  |  |  |  |  |  |  |  | | |
| Endline |  | 0.10 | -0.04 | 0.24 | 0.18 | 1.17 | -1.16 | 3.49 | 0.31 | 0.35 | -1.93 | 2.63 | 0.75 | n/a | n/a | n/a | n/a | | |
| Debris index (Mean, SD) |  |  |  |  |  |  |  |  |  |  |  |  |  |  |  |  |  | | |
| Baseline |  |  |  |  |  |  |  |  |  |  |  |  |  |  |  |  |  | | |
| Endline |  | -0.01 | -0.05 | 0.04 | 0.78 | <0.01 | -0.10 | 0.10 | 0.96 | 0.01 | -0.05 | 0.07 | 0.79 | -0.01 | -0.10 | 0.07 | 0.74 | | |
| Visited a dentist for pain or trouble with teeth (n, %) | | | | | | | | | | | | | | |  |  |  | | |
| Baseline |  |  |  |  |  |  |  |  |  |  |  |  |  |  |  |  |  | | |
| Endline |  | **0.24** | **0.10** | **0.57** | **<0.01** | 0.77 | 0.05 | 12.90 | 0.85 | 0.41 | 0.12 | 1.38 | 0.15 | **0.06** | **0.01** | **0.50** | **0.01** | | |
| Brush three times and more per day (n, %) | | | | | | | | | | | | | |  |  |  |  | | |
| Baseline |  |  |  |  |  |  |  |  |  |  |  |  |  |  |  |  |  | | |
| Endline |  | 1.96 | 0.88 | 4.36 | 0.10 | **11.63** | **1.02** | **132.08** | **0.05** | 1.92 | 0.61 | 6.11 | 0.26 | 1.11 | 0.24 | 5.10 | 0.90 | | |
| Brush for three minutes or more (n, %) |  |  |  |  |  |  |  |  |  |  |  |  |  |  |  |  |  | | |
| Baseline |  |  |  |  |  |  |  |  |  |  |  |  |  |  |  |  |  | | |
| Endline |  | **3.78** | **1.70** | **8.40** | **0.01** | **8.98** | **1.32** | **61.01** | **0.03** | 2.81 | 0.82 | 9.69 | 0.10 | 3.43 | 0.83 | 14.21 | 0.09 | | |
| Caregiver never care child's toothbrushing (n, %) | | | | | | | | | | | | | |  |  |  |  | | |
| Baseline |  |  |  |  |  |  |  |  |  |  |  |  |  |  |  |  |  | | |
| Endline |  | 3.24 | 0.56 | 18.70 | 0.19 | n/a | n/a | n/a | n/a | 1.37 | 0.18 | 10.69 | 0.76 | n/a | n/a | n/a | n/a | | |
|  |  |  |  |  |  |  |  |  |  |  |  |  |  |  |  |  |  | | |
| Adjusted with age, sex, time (baseline or endline), interaction of each variable and time for all participants' analyses. Age variable was not adjusted for the age stratified model. | | | | | | | | | | | | | | | | | |  |  |
| OHQOL: Oral health related quality of life, DMFT: Decayed, missing filled teeth | | | | | | | | | | | | | | | | |  | | |

**Appendix 8. Dentition type stratified longitudinal association between oral health outcomes and overall health outcomes changes in the intervention group**

| **1) Viral load detected ^a)^** |  |  |  |  |  |  |  |  |  |  |  |  |
| --- | --- | --- | --- | --- | --- | --- | --- | --- | --- | --- | --- | --- |
| Variables | All dentition types | | | | Mixed dentition at baseline | | | | Permanent teeth dentition at baseline | | | |
|  | Exp | 95% CI | | P Value | Exp | 95% CI | | P Value | Exp | 95% CI | | P Value |
| OHQOL | 0.62 | 0.28 | 1.38 | 0.25 | 1.04 | 0.38 | 2.83 | 0.94 | 0.28 | 0.07 | 1.15 | 0.08 |
| Time | 0.61 | 0.27 | 1.41 | 0.25 | 0.33 | 0.09 | 1.19 | 0.09 | 0.47 | 0.08 | 2.68 | 0.39 |
| OHQOL*Time | 0.98 | 0.29 | 3.31 | 0.97 | 1.45 | 0.29 | 7.35 | 0.65 | 1.05 | 0.13 | 8.30 | 0.96 |
|  |  |  |  |  |  |  |  |  |  |  |  |  |
| Salivary pH | 1.38 | 0.65 | 2.90 | 0.40 | 1.36 | 0.54 | 3.40 | 0.52 | 1.02 | 0.26 | 3.97 | 0.98 |
| Time | 0.74 | 0.33 | 1.66 | 0.46 | 0.62 | 0.20 | 1.97 | 0.41 | 0.34 | 0.06 | 1.88 | 0.87 |
| Salivary pH*Time | 0.52 | 0.16 | 1.67 | 0.27 | 0.39 | 0.08 | 1.94 | 0.25 | 1.18 | 0.16 | 8.43 | 0.87 |
|  |  |  |  |  |  |  |  |  |  |  |  |  |
| Salivary flow | 0.91 | 0.41 | 2.01 | 0.81 | 0.80 | 0.29 | 2.19 | 0.66 | 2.17 | 0.55 | 8.55 | 0.27 |
| Time | 0.49 | 0.23 | 1.07 | 0.07 | 0.43 | 0.15 | 1.24 | 0.12 | 0.25 | 0.03 | 1.79 | 0.17 |
| Salivary flow*Time | 1.25 | 0.40 | 3.94 | 0.71 | 0.79 | 0.18 | 3.42 | 0.75 | 1.29 | 0.16 | 10.29 | 0.81 |
|  |  |  |  |  |  |  |  |  |  |  |  |  |
| DMFT/dmft | 0.59 | 0.25 | 1.37 | 0.22 | 1.11 | 0.35 | 3.51 | 0.86 | 0.43 | 0.11 | 1.77 | 0.24 |
| Time | 0.64 | 0.31 | 1.33 | 0.23 | 0.74 | 0.25 | 2.19 | 0.59 | 0.38 | 0.07 | 2.10 | 0.27 |
| DMFT/dmft*Time | 0.75 | 0.23 | 2.46 | 0.63 | 0.30 | 0.03 | 1.53 | 0.14 | 1.23 | 0.16 | 9.64 | 0.85 |
|  |  |  |  |  |  |  |  |  |  |  |  |  |
| DMFT | 0.71 | 0.29 | 1.78 | 0.47 | 0.45 | 0.09 | 2.21 | 0.33 | 1.78 | 0.43 | 7.31 | 0.42 |
| Time | 0.31 | 0.14 | 0.68 | <0.01 | 0.28 | 0.10 | 0.78 | 0.02 | 0.25 | 0.03 | 2.07 | 0.20 |
| DMFT*Time | 3.58 | 1.10 | 11.73 | 0.04 | 4.35 | 0.83 | 22.75 | 0.08 | 2.09 | 0.24 | 17.87 | 0.50 |
|  |  |  |  |  |  |  |  |  |  |  |  |  |
| Debris index | 1.57 | 0.69 | 3.61 | 0.28 | 4.12 | 0.96 | 17.67 | 0.06 | 1.45 | 0.36 | 5.85 | 0.60 |
| Time | 0.70 | 0.31 | 1.58 | 0.39 | 1.12 | 0.25 | 4.98 | 0.88 | 0.42 | 0.09 | 1.91 | 0.26 |
| Debris index*Time | 0.86 | 0.16 | 4.52 | 0.86 | 0.44 | 0.06 | 3.18 | 0.41 | n/a | n/a | n/a | n/a |
|  |  |  |  |  |  |  |  |  |  |  |  |  |
| Adjusted with age, sex, time (baseline or endline), interaction of each variable and time for all participants' analyses. | | | | | | | | | | | | |
| OHQOL: Oral health related quality of life, DMFT: Decayed, missing filled teeth | | | | | | | | | | | | |
| Time: Endline=1, Baseline =0 | | | | | | | | | | | | |
| 1. Binary values with a mean cut off was used for the analysis: above mean=1, below mean=0 | | | | | | | | | | | | |
|  |  |  |  |  |  |  |  |  |  |  |  |  |
| **2) Height for age** |  |  |  |  |  |  |  |  |  |  |  |  |
| Variables | All dentition types | | | | Mixed dentition at baseline | | | | Permanent teeth dentition at baseline | | | |
|  | Estimate | 95% CI | | P Value | Exp | 95% CI | | P Value | Exp | 95% CI | | P Value |
| OHQOL | -0.05 | -0.12 | 0.03 | 0.23 | -0.08 | -0.22 | 0.06 | 0.26 | -0.01 | -0.08 | 0.06 | 0.75 |
| Time | -0.01 | -0.14 | 0.12 | 0.85 | -0.07 | -0.31 | 0.18 | 0.60 | 0.02 | -0.11 | 0.15 | 0.80 |
| OHQOL*Time | 0.02 | -0.08 | 0.12 | 0.69 | 0.05 | -0.13 | 0.24 | 0.57 | 0.01 | -0.09 | 0.11 | 0.87 |
|  |  |  |  |  |  |  |  |  |  |  |  |  |
| Salivary pH | 0.38 | 0.11 | 0.64 | 0.01 | 0.76 | 0.32 | 1.20 | <0.01 | -0.04 | -0.30 | 0.23 | 0.79 |
| Time | 0.46 | 0.18 | 0.74 | <0.01 | 0.80 | 0.35 | 1.25 | <0.01 | -0.07 | -0.37 | 0.22 | 0.62 |
| Salivary pH*Time | -0.57 | -0.92 | -0.21 | <0.01 | -1.01 | -1.58 | -0.44 | <0.01 | 0.13 | -0.25 | 0.51 | 0.50 |
|  |  |  |  |  |  |  |  |  |  |  |  |  |
| Salivary flow | 0.02 | -0.03 | 0.07 | 0.40 | 0.02 | -0.07 | 0.10 | 0.73 | 0.02 | -0.04 | 0.07 | 0.53 |
| Time | 0.03 | -0.01 | 0.06 | 0.13 | 0.02 | -0.05 | 0.08 | 0.58 | 0.03 | -0.01 | 0.07 | 0.17 |
| Salivary flow*Time | -0.02 | -0.09 | 0.04 | 0.48 | -0.02 | -0.13 | 0.10 | 0.78 | -0.01 | -0.08 | 0.06 | 0.85 |
|  |  |  |  |  |  |  |  |  |  |  |  |  |
| DMFT/dmft | -0.02 | -0.06 | 0.02 | 0.44 | -0.09 | -0.18 | -0.01 | 0.03 | 0.03 | -0.01 | 0.07 | 0.11 |
| Time | 0.02 | -0.02 | 0.05 | 0.42 | -0.04 | -0.12 | 0.05 | 0.39 | 0.04 | <0.01 | 0.07 | 0.05 |
| DMFT/dmft*Time | <0.01 | -0.04 | 0.04 | 0.98 | 0.04 | -0.04 | 0.13 | 0.33 | -0.01 | -0.05 | 0.03 | 0.61 |
|  |  |  |  |  |  |  |  |  |  |  |  |  |
| DMFT | -0.01 | -0.05 | 0.03 | 0.66 | -0.05 | -0.12 | 0.02 | 0.16 | 0.03 | -0.01 | 0.07 | 0.11 |
| Time | 0.01 | -0.02 | 0.04 | 0.41 | -0.01 | -0.07 | 0.05 | 0.81 | 0.04 | <0.01 | 0.07 | 0.05 |
| DMFT*Time | <0.01 | -0.04 | 0.05 | 0.85 | 0.03 | -0.05 | 0.11 | 0.51 | -0.01 | -0.05 | 0.03 | 0.61 |
|  |  |  |  |  |  |  |  |  |  |  |  |  |
| Debris index | -0.05 | -0.15 | 0.05 | 0.32 | -0.12 | -0.31 | 0.08 | 0.24 | -0.06 | -0.15 | 0.03 | 0.20 |
| Time | 0.01 | -0.04 | 0.06 | 0.82 | <0.01 | -0.11 | 0.10 | 0.97 | <0.01 | -0.05 | 0.04 | 0.93 |
| Debris index*Time | <0.01 | -0.12 | 0.12 | 1.00 | -0.03 | -0.25 | 0.20 | 0.82 | 0.10 | -0.02 | 0.22 | 0.09 |
| Adjusted with age, sex, time (baseline or endline), interaction of each variable and time for all participants' analyses. Age variable was not adjusted for the age stratified model. | | | | | | | | | | | | |
| OHQOL: Oral health related quality of life, DMFT: Decayed, missing filled teeth | | | | | | | | | | | | |
| Time: Endline=1, Baseline =0 | | | | | | | | | | | | |

|  |  |  |  |  |  |  |  |  |  |  |  |  |
| --- | --- | --- | --- | --- | --- | --- | --- | --- | --- | --- | --- | --- |
| **3) Body-mass-index for age** |  |  |  |  |  |  |  |  |  |  |  |  |
| Variables | All dentition types | | | | Mixed dentition at baseline | | | | Permanent teeth dentition at baseline | | | |
|  | Estimate | 95% CI | | P Value | Exp | 95% CI | | P Value | Exp | 95% CI | | P Value |
| OHQOL | 0.05 | -0.05 | 0.16 | 0.97 | 0.11 | -0.07 | 0.28 | 0.23 | 0.01 | -0.11 | 0.14 | 0.82 |
| Time | -0.44 | -0.63 | -0.25 | <0.01 | -0.55 | -0.86 | -0.24 | <0.01 | -0.22 | -0.45 | 0.02 | 0.07 |
| OHQOL*Time | 0.07 | -0.08 | 0.22 | 0.34 | 0.16 | -0.07 | 0.40 | 0.17 | -0.09 | -0.28 | 0.09 | 0.33 |
|  |  |  |  |  |  |  |  |  |  |  |  |  |
| Salivary pH | 0.11 | -0.26 | 0.48 | 0.57 | 0.12 | -0.48 | 0.71 | 0.70 | -1.03 | -0.46 | 0.40 | 0.90 |
| Time | -0.91 | -1.31 | -0.51 | <0.01 | -0.59 | -1.23 | 0.05 | 0.07 | -1.37 | -1.87 | -0.88 | <0.01 |
| Salivary pH*Time | 0.71 | 0.20 | 1.23 | 0.01 | 0.30 | -0.52 | 1.12 | 0.47 | 1.33 | 0.70 | 1.96 | <0.01 |
|  |  |  |  |  |  |  |  |  |  |  |  |  |
| Salivary flow | 0.04 | -0.03 | 0.11 | 0.26 | 0.07 | -0.04 | 0.17 | 0.24 | 0.01 | -0.09 | 0.10 | 0.89 |
| Time | -0.39 | -0.44 | -0.34 | <0.01 | -0.35 | -0.44 | -0.27 | <0.01 | -0.42 | -0.50 | -0.35 | <0.01 |
| Salivary flow*Time | 0.09 | -0.01 | 0.18 | 0.08 | <0.01 | -0.16 | 0.15 | 0.96 | 0.17 | 0.04 | 0.29 | 0.01 |
|  |  |  |  |  |  |  |  |  |  |  |  |  |
| DMFT/dmft | -0.03 | -0.08 | 0.03 | 0.31 | -0.07 | -0.17 | 0.03 | 0.18 | <0.01 | -0.06 | 0.07 | 0.95 |
| Time | -0.30 | -0.35 | -0.24 | <0.01 | -0.31 | -0.42 | -0.20 | <0.01 | -0.30 | -0.37 | -0.23 | <0.01 |
| DMFT/dmft*Time | -0.07 | -0.14 | -0.01 | 0.03* | -0.07 | -0.19 | 0.05 | 0.23 | -0.05 | -0.13 | 0.03 | 0.19 |
|  |  |  |  |  |  |  |  |  |  |  |  |  |
| DMFT | -0.01 | -0.06 | 0.05 | 0.83 | 0.01 | -0.07 | 0.10 | 0.76 | <0.01 | -0.06 | 0.07 | 0.95 |
| Time | -0.32 | -0.36 | -0.27 | <0.01 | -0.29 | -0.36 | -0.21 | <0.01 | -0.30 | -0.37 | -0.23 | <0.01 |
| DMFT*Time | -0.06 | -0.12 | <0.01 | 0.06 | -0.12 | -0.23 | -0.01 | 0.03 | -0.05 | -0.13 | 0.03 | 0.19 |
|  |  |  |  |  |  |  |  |  |  |  |  |  |
| Debris index | -0.04 | -0.18 | 0.10 | 0.55 | -0.01 | -0.26 | 0.23 | 0.91 | -0.05 | -0.22 | 0.12 | 0.55 |
| Time | -0.31 | -0.38 | -0.24 | <0.01 | -0.28 | -0.41 | -0.15 | <0.01 | -0.31 | -0.39 | -0.23 | <0.01 |
| Debris index*Time | -0.20 | -0.36 | -0.04 | 0.02 | -0.25 | -0.54 | 0.05 | 0.10 | -0.15 | -0.38 | 0.08 | 0.19 |
| Adjusted with age, sex, time (baseline or endline), interaction of each variable and time for all participants' analyses. Age variable was not adjusted for the age stratified model. | | | | | | | | | | | | |
| OHQOL: Oral health related quality of life, DMFT: Decayed, missing filled teeth | | | | | | | | | | | | |
| Time: Endline=1, Baseline =0 | | | | | | | | | | | | |

|  |  |  |  |  |  |  |  |  |  |  |  |  |
| --- | --- | --- | --- | --- | --- | --- | --- | --- | --- | --- | --- | --- |
| **4) Overall quality of life** |  |  |  |  |  |  |  |  |  |  |  |  |
| Variables | All dentition types | | | | Mixed dentition at baseline | | | | Permanent teeth dentition at baseline | | | |
|  | Estimate | 95% CI | | P Value | Exp | 95% CI | | P Value | Exp | 95% CI | | P Value |
| OHQOL | -0.22 | -0.28 | -0.16 | <0.01 | -0.19 | -0.29 | -0.09 | <0.01 | -0.25 | -0.33 | -0.18 | <0.01 |
| Time | -0.04 | -0.15 | 0.07 | 0.48 | 0.01 | -0.17 | 0.19 | 0.94 | -0.06 | -0.21 | 0.10 | 0.47 |
| OHQOL*Time | 0.05 | -0.04 | 0.13 | 0.28 | 0.02 | -0.12 | 0.15 | 0.81 | 0.04 | -0.08 | 0.16 | 0.47 |
|  |  |  |  |  |  |  |  |  |  |  |  |  |
| Salivary pH | -0.03 | -0.26 | 0.20 | 0.79 | 0.04 | -0.33 | 0.40 | 0.84 | -0.01 | -0.36 | 0.35 | 0.99 |
| Time | 0.13 | -0.14 | 0.39 | 0.36 | 0.14 | -0.26 | 0.55 | 0.48 | 0.22 | -0.20 | 0.65 | 0.30 |
| Salivary pH*Time | -0.11 | -0.45 | 0.23 | 0.52 | -0.12 | -0.64 | 0.39 | 0.64 | -0.25 | -0.79 | 0.29 | 0.36 |
|  |  |  |  |  |  |  |  |  |  |  |  |  |
| Salivary flow | -0.06 | -0.11 | -0.02 | 0.01 | -0.10 | -0.16 | -0.03 | <0.01 | -0.02 | -0.10 | 0.05 | 0.55 |
| Time | 0.01 | -0.03 | 0.04 | 0.73 | 0.01 | -0.04 | 0.06 | 0.73 | 0.01 | -0.06 | 0.07 | 0.84 |
| Salivary flow*Time | 0.07 | 0.01 | 0.13 | 0.03 | 0.08 | -0.01 | 0.18 | 0.09 | 0.03 | -0.07 | 0.14 | 0.53 |
|  |  |  |  |  |  |  |  |  |  |  |  |  |
| DMFT/dmft | <-0.01 | -0.04 | 0.03 | 0.68 | 0.01 | -0.05 | 0.07 | 0.70 | -0.01 | -0.06 | 0.04 | 0.74 |
| Time | 0.04 | <-0.01 | 0.08 | 0.05 | 0.06 | -0.01 | 0.13 | 0.08 | 0.02 | -0.03 | 0.08 | 0.44 |
| DMFT/dmft*Time | <-0.01 | -0.05 | 0.04 | 0.93 | -0.02 | -0.10 | 0.06 | 0.65 | <0.01 | -0.07 | 0.07 | 0.99 |
|  |  |  |  |  |  |  |  |  |  |  |  |  |
| DMFT | 0.14 | -0.02 | 0.05 | 0.40 | 0.03 | -0.02 | 0.09 | 0.24 | -0.01 | -0.06 | 0.04 | 0.74 |
| Time | 0.04 | 0.01 | 0.07 | 0.01 | 0.05 | <0.01 | 0.10 | 0.05 | 0.02 | -0.03 | 0.08 | 0.44 |
| DMFT*Time | -0.01 | -0.05 | 0.03 | 0.67 | <0.01 | -0.08 | 0.07 | 0.91 | <0.01 | -0.07 | 0.07 | 0.99 |
|  |  |  |  |  |  |  |  |  |  |  |  |  |
| Debris index | <0.01 | -0.09 | 0.09 | 0.92 | -0.09 | -0.24 | 0.06 | 0.26 | 0.11 | -0.01 | 0.23 | 0.08 |
| Time | 0.03 | -0.02 | 0.08 | 0.25 | -0.02 | -0.11 | 0.06 | 0.57 | 0.07 | <0.01 | 0.13 | 0.04 |
| Debris index*Time | 0.04 | -0.07 | 0.15 | 0.46 | 0.18 | -0.01 | 0.37 | 0.06 | -0.09 | -0.27 | 0.08 | 0.29 |
| Adjusted with age, sex, time (baseline or endline), interaction of each variable and time for all participants' analyses. Age variable was not adjusted for the age stratified model. | | | | | | | | | | | | |
| OHQOL: Oral health related quality of life, DMFT: Decayed, missing filled teeth | | | | | | | | | | | | |
| Time: Endline=1, Baseline =0 | | | | | | | | | | | | |

**Appendix 9. Age group stratified longitudinal association between oral health outcomes and overall health outcomes changes in the intervention group**

| 1. **Viral load detected ^a)^** |  |  | | |  | |  | | | |  | | |  |  | | |  | |  |  | |  | | |  | |  | | |  | |  | |  |
| --- | --- | --- | --- | --- | --- | --- | --- | --- | --- | --- | --- | --- | --- | --- | --- | --- | --- | --- | --- | --- | --- | --- | --- | --- | --- | --- | --- | --- | --- | --- | --- | --- | --- | --- | --- |
| Variables | All age groups | | | | | | | | | 6-8 years old at baseline | | | | | | | | | 9-12 years old at baseline | | | | | | | | 13-15 years old at baseline | | | | | | | | |
|  | Exp | | | 95% CI | | | | | P Value | Exp | | | 95% CI | | | | P Value | | Exp | 95% CI | | | | P Value | | | Exp | | 95% CI | | | | | P Value | |
| OHQOL | 0.62 | | 0.28 | | | 1.38 | | 0.25 | | 0.42 | | 0.05 | | | | 3.55 | 0.42 | | 0.41 | 0.12 | | 1.33 | | | 0.14 | | 0.61 | | | 0.14 | | 2.56 | | | 0.49 |
| Time | 0.61 | | 0.27 | | | 1.41 | | 0.25 | | 0.06 | | 0.01 | | | | 0.53 | 0.01 | | 0.46 | 0.14 | | 1.59 | | | 0.22 | | 1.17 | | | 0.28 | | 4.82 | | | 0.83 |
| OHQOL*Time | 0.98 | | 0.29 | | | 3.31 | | 0.97 | | 8.48 | | 0.16 | | | | 456.19 | 0.28 | | 0.94 | 0.14 | | 6.19 | | | 0.95 | | 0.93 | | | 0.12 | | 7.26 | | | 0.94 |
|  |  | |  | | |  | |  | |  | |  | | | |  |  | |  |  | |  | | |  | |  | | |  | |  | | |  |
| Salivary pH | 1.38 | | 0.65 | | | 2.90 | | 0.40 | | 2.00 | | 0.30 | | | | 13.59 | 0.47 | | 1.05 | 0.33 | | 3.33 | | | 0.93 | | 2.12 | | | 0.51 | | 8.87 | | | 0.30 |
| Time | 0.74 | | 0.33 | | | 1.66 | | 0.46 | | 0.24 | | 0.03 | | | | 1.89 | 0.17 | | 0.36 | 0.10 | | 1.30 | | | 0.12 | | 1.39 | | | 0.37 | | 5.16 | | | 0.62 |
| Salivary pH*Time | 0.52 | | 0.16 | | | 1.67 | | 0.27 | | 0.32 | | 0.02 | | | | 6.91 | 0.46 | | 0.73 | 0.10 | | 5.10 | | | 0.75 | | 0.52 | | | 0.08 | | 3.62 | | | 0.50 |
|  |  | |  | | |  | |  | |  | |  | | | |  |  | |  |  | |  | | |  | |  | | |  | |  | | |  |
| Salivary flow | 0.91 | | 0.41 | | | 2.01 | | 0.81 | | 0.41 | | 0.04 | | | | 3.99 | 0.43 | | 0.56 | 0.15 | | 2.13 | | | 0.39 | | 2.78 | | | 0.68 | | 11.39 | | | 0.15 |
| Time | 0.49 | | 0.23 | | | 1.07 | | 0.07 | | 0.09 | | 0.01 | | | | 0.60 | 0.02 | | 0.45 | 0.15 | | 1.35 | | | 0.15 | | 0.89 | | | 0.20 | | 3.99 | | | 0.88 |
| Salivary flow*Time | 1.25 | | 0.40 | | | 3.94 | | 0.71 | | 3.23 | | 0.13 | | | | 81.90 | 0.46 | | 0.39 | 0.04 | | 3.90 | | | 0.42 | | 0.96 | | | 0.13 | | 7.18 | | | 0.97 |
|  |  | |  | | |  | |  | |  | |  | | | |  |  | |  |  | |  | | |  | |  | | |  | |  | | |  |
| DMFT/dmft | 0.59 | | 0.25 | | | 1.37 | | 0.22 | | n/a | | n/a | | | | n/a | n/a | | 0.84 | 0.23 | | 3.00 | | | 0.78 | | 0.57 | | | 0.13 | | 2.42 | | | 0.44 |
| Time | 0.64 | | 0.31 | | | 1.33 | | 0.23 | | n/a | | n/a | | | | n/a | n/a | | 0.41 | 0.12 | | 1.35 | | | 0.14 | | 1.90 | | | 0.49 | | 7.36 | | | 0.34 |
| DMFT/dmft*Time | 0.75 | | 0.23 | | | 2.46 | | 0.63 | | n/a | | n/a | | | | n/a | n/a | | 0.59 | 0.08 | | 4.59 | | | 0.61 | | 0.47 | | | 0.07 | | 3.23 | | | 0.44 |
|  |  | |  | | |  | |  | |  | |  | | | |  |  | |  |  | |  | | |  | |  | | |  | |  | | |  |
| DMFT | 0.71 | | 0.29 | | | 1.78 | | 0.47 | | 1.11 | | 0.27 | | | | 4.60 | 0.88 | | 0.77 | 0.21 | | 2.76 | | | 0.68 | | 1.11 | | | 0.27 | | 4.60 | | | 0.88 |
| Time | 0.31 | | 0.14 | | | 0.68 | | <0.01 | | 0.68 | | 0.17 | | | | 2.63 | 0.57 | | 0.16 | 0.04 | | 0.65 | | | 0.01 | | 0.68 | | | 0.17 | | 2.63 | | | 0.57 |
| DMFT*Time | 3.58 | | 1.10 | | | 11.73 | | 0.04 | | 2.76 | | 0.41 | | | | 18.80 | 0.29 | | 3.74 | 0.56 | | 24.95 | | | 0.17 | | 2.76 | | | 0.41 | | 18.80 | | | 0.29 |
|  |  | |  | | |  | |  | |  | |  | | | |  |  | |  |  | |  | | |  | |  | | |  | |  | | |  |
| Debris index | 1.57 | | 0.69 | | | 3.61 | | 0.28 | | 0.70 | | 0.02 | | | | 22.14 | 0.84 | | 4.72 | 0.67 | | 33.36 | | | 0.12 | | 0.66 | | | 0.15 | | 3.00 | | | 0.59 |
| Time | 0.70 | | 0.31 | | | 1.58 | | 0.39 | | 0.04 | | <0.01 | | | | 1.71 | 0.09 | | 1.14 | 0.17 | | 7.72 | | | 0.89 | | 0.88 | | | 0.30 | | 2.52 | | | 0.80 |
| Debris index*Time | 0.86 | | 0.16 | | | 4.52 | | 0.86 | | 45.33 | | 0.31 | | | | 6593.28 | 0.13 | | 0.24 | 0.01 | | 4.94 | | | 0.35 | | n/a | | | n/a | | n/a | | | n/a |
| Adjusted with age, sex, time (baseline or endline), interaction of each variable and time for all participants' analyses. Age variable was not adjusted for the age stratified model. | | | | | | | | | | | | | | | | | | | | | | | | | | | | | | | | | | | |
| OHQOL: Oral health related quality of life, DMFT: Decayed, missing filled teeth | | | | | | | | | | | | | | | | | | | | | | | | | | | | | | | | | | | |
| Time: Endline=1, Baseline =0 |  |  | | |  | |  | | | |  | | |  |  | | |  | |  |  | |  | | |  | |  | | |  | |  | |  |
| Binary values with a mean cut off was used for the analysis: above mean=1, below mean=0 | | | | | | | | | | | | | | | | | | | | | | | | | | | | | | |  | |  | |  |

| 1. **Height for age** |  |  | | |  | |  | |  | |  | |  | | |  | |  | |  | | | |  | |  |  | | |  | |  | | |  |  |
| --- | --- | --- | --- | --- | --- | --- | --- | --- | --- | --- | --- | --- | --- | --- | --- | --- | --- | --- | --- | --- | --- | --- | --- | --- | --- | --- | --- | --- | --- | --- | --- | --- | --- | --- | --- | --- |
| Variables | All age groups | | | | | | | | 6-8 years old at baseline | | | | | | | | | 9-12 years old at baseline | | | | | | 13-15 years old at baseline | | | | | | | | | | | | |
|  | Estimate | 95% CI | | | | | P Value | | Exp | | 95% CI | | | P Value | | | | Exp | | 95% CI | | P Value | | Exp | | | | 95% CI | | | | | P Value | | | |
| OHQOL | -0.05 | | -0.12 | 0.03 | | 0.23 | | 0.47 | | 0.85 | | 0.10 | | | 0.02 | | <0.01 | | -0.08 | | 0.09 | | 0.94 | | 0.02 | | | | -0.06 | | 0.10 | | | 0.56 | | |
| Time | -0.01 | | -0.14 | 0.12 | | 0.85 | | 0.58 | | 1.28 | | 0.10 | | | 0.09 | | 0.12 | | -0.02 | | 0.26 | | 0.09 | | <0.01 | | | | -0.15 | | 0.15 | | | 0.96 | | |
| OHQOL*Time | 0.02 | | -0.08 | 0.12 | | 0.69 | | 0.46 | | -0.06 | | 0.98 | | | 0.08 | | -0.09 | | -0.21 | | 0.02 | | 0.10 | | 0.02 | | | | -0.10 | | 0.14 | | | 0.71 | | |
|  |  | |  |  | |  | |  | |  | |  | | |  | |  | |  | |  | |  | |  | | | |  | |  | | |  | | |
| Salivary pH | 0.38 | | 0.11 | 0.64 | | 0.01 | | 1.37 | | 0.37 | | 2.37 | | | 0.01 | | -0.05 | | -0.29 | | 0.20 | | 0.71 | | -0.01 | | | | -0.31 | | 0.29 | | | 0.97 | | |
| Time | 0.46 | | 0.18 | 0.74 | | <0.01 | | 0.17 | | -0.10 | | 0.14 | | | 0.77 | | 0.05 | | 0.01 | | 0.09 | | 0.016 | | 0.02 | | | | -0.02 | | 0.06 | | | 0.04 | | |
| Salivary pH*Time | -0.57 | | -0.92 | -0.21 | | <0.01 | | -0.15 | | -0.39 | | 0.08 | | | 0.20 | | -0.01 | | -0.08 | | 0.07 | | 0.86 | | 0.02 | | | | -0.06 | | 0.10 | | | 0.59 | | |
|  |  | |  |  | |  | |  | |  | |  | | |  | |  | |  | |  | |  | |  | | | |  | |  | | |  | | |
| Salivary flow | 0.02 | | -0.03 | 0.07 | | 0.40 | | -0.01 | | -0.12 | | 0.11 | | | 0.93 | | 0.05 | | 0.01 | | 0.09 | | 0.02 | | 0.02 | | | | -0.02 | | 0.06 | | | 0.35 | | |
| Time | 0.03 | | -0.01 | 0.06 | | 0.13 | | -0.12 | | -0.33 | | 0.08 | | | 0.23 | | 0.08 | | 0.01 | | 0.14 | | 0.02 | | 0.02 | | | | -0.05 | | 0.08 | | | 0.60 | | |
| Salivary flow*Time | -0.02 | | -0.09 | 0.04 | | 0.48 | | 0.08 | | -0.19 | | 0.36 | | | 0.54 | | -0.09 | | -0.16 | | -0.01 | | 0.03 | | <0.01 | | | | -0.08 | | 0.08 | | | 0.93 | | |
|  |  | |  |  | |  | |  | |  | |  | | |  | |  | |  | |  | |  | |  | | | |  | |  | | |  | | |
| DMFT/dmft | -0.02 | | -0.06 | 0.02 | | 0.44 | | -0.20 | | -0.46 | | 0.06 | | | 0.12 | | 0.05 | | 0.09 | | 0.01 | | 0.02 | | 0.06 | | | | 0.02 | | 0.11 | | | 0.01 | | |
| Time | 0.02 | | -0.02 | 0.05 | | 0.42 | | 0.15 | | 0.47 | | 0.16 | | | 0.33 | | <0.01 | | -0.04 | | 0.04 | | 0.94 | | 0.04 | | | | 0.01 | | 0.07 | | | 0.02 | | |
| DMFT/dmft*Time | <0.01 | | -0.04 | 0.04 | | 0.98 | | 0.15 | | -0.16 | | 0.47 | | | 0.33 | | 0.01 | | -0.05 | | 56.00 | | 0.84 | | -0.02 | | | | -0.06 | | 0.02 | | | 0.35 | | |
|  |  | |  |  | |  | |  | |  | |  | | |  | |  | |  | |  | |  | |  | | | |  | |  | | |  | | |
| DMFT | -0.01 | | -0.05 | 0.03 | | 0.66 | | -0.08 | | -0.30 | | 0.14 | | | 0.46 | | -0.04 | | -0.08 | | 0.01 | | 0.13 | | 0.07 | | | | 0.03 | | 0.12 | | | <0.01 | | |
| Time | 0.01 | | -0.02 | 0.04 | | 0.41 | | -0.02 | | -0.14 | | 0.11 | | | 0.77 | | 0.01 | | -0.03 | | 0.04 | | 0.75 | | 0.04 | | | | 0.01 | | 0.07 | | | 0.01 | | |
| DMFT*Time | <0.01 | | -0.04 | 0.05 | | 0.85 | | 0.09 | | -0.18 | | 0.36 | | | 0.50 | | 0.01 | | -0.04 | | 0.06 | | 0.66 | | -0.03 | | | | -0.07 | | 0.02 | | | 0.23 | | |
|  |  | |  |  | |  | |  | |  | |  | | |  | |  | |  | |  | |  | |  | | | |  | |  | | |  | | |
| Debris index | -0.05 | | -0.15 | 0.05 | | 0.32 | | -0.09 | | -1.03 | | 0.85 | | | 0.85 | | -0.04 | | -0.20 | | 0.12 | | 0.64 | | -0.08 | | | | -0.16 | | <0.01 | | | 0.05 | | |
| Time | 0.01 | | -0.04 | 0.06 | | 0.82 | | 0.01 | | -0.54 | | 0.55 | | | 0.98 | | 0.02 | | -0.06 | | 0.10 | | 0.68 | | -0.03 | | | | -0.07 | | 0.01 | | | 0.14 | | |
| Debris index*Time | <0.01 | | -0.12 | 0.12 | | 1.00 | | -0.04 | | -1.11 | | 1.03 | | | 0.94 | | -0.06 | | -0.22 | | 0.11 | | 0.51 | | 0.19 | | | | 0.05 | | 0.32 | | | 0.01 | | |
| Adjusted with age, sex, time (baseline or endline), interaction of each variable and time for all participants' analyses. Age variable was not adjusted for the age stratified model. | | | | | | | | | | | | | | | | | | | | | | | | | | | | | | | | | | | | |
| OHQOL: Oral health related quality of life, DMFT: Decayed, missing filled teeth | | | | | | | | | | | | | | | | | | | | | | | |  | |  |  | | |  | |  | | |  |  |
| Time: Endline=1, Baseline =0 | | | | | | | | | | | | | | | | | | | | | | | |  | |  |  | | |  | |  | | |  |  |

| 1. **Body-mass-index for age** | | | | | | |  | | | |  | | |  | |  | | | | |  | | |  | |  | |  | | | | |  |  | |  |  | | |  |
| --- | --- | --- | --- | --- | --- | --- | --- | --- | --- | --- | --- | --- | --- | --- | --- | --- | --- | --- | --- | --- | --- | --- | --- | --- | --- | --- | --- | --- | --- | --- | --- | --- | --- | --- | --- | --- | --- | --- | --- | --- |
| Variables | All age groups | | | | | | | | | 6-8 years old at baseline | | | | | | | | | | 9-12 years old at baseline | | | | | | | | | | 13-15 years old at baseline | | | | | | | | | | |
|  | Estimate | | 95% CI | | P Value | | | | Exp | | | | 95% CI | | | | P Value | | | Exp | | 95% CI | | | | | P Value | | | Exp | 95% CI | | | | | | | | P Value | |
| OHQOL | 0.05 | -0.05 | | 0.16 | | 0.97 | | 0.22 | | | | 0.21 | | | 0.65 | | | 0.31 | 0.04 | | | | -0.12 | | 0.19 | | 0.64 | | <0.01 | | | -0.14 | | | 0.14 | | | 0.98 | | |
| Time | -0.44 | -0.63 | | -0.25 | | <0.01 | | -0.57 | | | | -1.35 | | | 0.21 | | | 0.15 | -0.44 | | | | -0.71 | | -0.17 | | <0.01 | | -0.37 | | | -0.63 | | | -0.10 | | | 0.01 | | |
| OHQOL*Time | 0.07 | -0.08 | | 0.22 | | 0.34 | | 0.15 | | | | -0.45 | | | 0.75 | | | 0.61 | 0.08 | | | | -0.14 | | 0.29 | | 0.48 | | 0.02 | | | -0.19 | | | 0.23 | | | 0.87 | | |
|  |  |  | |  | |  | |  | | | |  | | |  | | |  |  | | | |  | |  | |  | |  | | |  | | |  | | |  | | |
| Salivary pH | 0.11 | -0.26 | | 0.48 | | 0.57 | | -0.75 | | | | -2.10 | | | 0.61 | | | 0.27 | 0.23 | | | | -0.28 | | 0.73 | | 0.37 | | 0.30 | | | -0.22 | | | 0.82 | | | 0.26 | | |
| Time | -0.91 | -1.31 | | -0.51 | | <0.01 | | -1.89 | | | | -3.36 | | | -0.43 | | | 0.01 | -0.45 | | | | -1.05 | | 0.16 | | 0.14 | | -1.11 | | | -1.63 | | | -0.58 | | | <0.01 | | |
| Salivary pH*Time | 0.71 | 0.20 | | 1.23 | | 0.01 | | 1.92 | | | | 0.06 | | | 3.79 | | | 0.04 | 0.12 | | | | -0.65 | | 0.88 | | 0.76 | | 0.97 | | | 0.30 | | | 1.63 | | | 0.01 | | |
|  |  |  | |  | |  | |  | | | |  | | |  | | |  |  | | | |  | |  | |  | |  | | |  | | |  | | |  | | |
| Salivary flow | 0.04 | -0.03 | | 0.11 | | 0.26 | | 0.11 | | | | -0.11 | | | 0.33 | | | 0.34 | 0.02 | | | | -0.09 | | 0.13 | | 0.70 | | 0.04 | | | -0.06 | | | 0.14 | | | 0.41 | | |
| Time | -0.39 | -0.44 | | -0.34 | | <0.01 | | -0.38 | | | | -0.52 | | | -0.23 | | | <0.01 | -0.40 | | | | -0.48 | | -0.32 | | <0.01 | | -0.41 | | | -0.48 | | | -0.35 | | | <0.01 | | |
| Salivary flow*Time | 0.09 | -0.01 | | 0.18 | | 0.08 | | -0.02 | | | | -0.36 | | | 0.31 | | | 0.88 | 0.09 | | | | -0.07 | | 0.24 | | 0.26 | | 0.12 | | | -0.01 | | | 0.26 | | | 0.07 | | |
|  |  |  | |  | |  | |  | | | |  | | |  | | |  |  | | | |  | |  | |  | |  | | |  | | |  | | |  | | |
| DMFT/dmft | -0.03 | -0.08 | | 0.03 | | 0.31 | | -0.01 | | | | -0.29 | | | 0.26 | | | 0.92 | -0.01 | | | | -0.08 | | 0.06 | | 0.72 | | -0.05 | | | -0.13 | | | 0.03 | | | 0.21 | | |
| Time | -0.30 | -0.35 | | -0.24 | | <0.01 | | -0.22 | | | | -0.58 | | | 0.14 | | | 0.23 | -0.27 | | | | -0.35 | | -0.19 | | <0.01 | | -0.35 | | | -0.41 | | | -0.29 | | | <0.01 | | |
| DMFT/dmft*Time | -0.07 | -0.14 | | -0.01 | | 0.03 | | -0.19 | | | | -0.55 | | | 0.17 | | | 0.29 | -0.12 | | | | -0.22 | | -0.03 | | 0.01 | | <0.01 | | | -0.08 | | | 0.08 | | | 0.97 | | |
|  |  |  | |  | |  | |  | | | |  | | |  | | |  |  | | | |  | |  | |  | |  | | |  | | |  | | |  | | |
| DMFT | -0.01 | -0.06 | | 0.05 | | 0.83 | | 0.16 | | | | -0.08 | | | 0.39 | | | 0.19 | -0.23 | | | | -0.09 | | 0.05 | | 0.52 | | -0.03 | | | -0.11 | | | 0.05 | | | 0.48 | | |
| Time | -0.32 | -0.36 | | -0.27 | | <0.01 | | -0.31 | | | | -0.45 | | | -0.16 | | | <0.01 | -0.29 | | | | -0.35 | | -0.22 | | <0.01 | | -0.34 | | | -0.40 | | | -0.28 | | | <0.01 | | |
| DMFT*Time | -0.06 | -0.12 | | <0.01 | | 0.06 | | -0.21 | | | | -0.52 | | | 0.10 | | | 0.17 | -0.10 | | | | -0.19 | | -0.01 | | 0.04 | | -0.01 | | | -0.10 | | | 0.07 | | | 0.73 | | |
|  |  |  | |  | |  | |  | | | |  | | |  | | |  |  | | | |  | |  | |  | |  | | |  | | |  | | |  | | |
| Debris index | -0.04 | -0.18 | | 0.10 | | 0.55 | | -0.02 | | | | -1.00 | | | 0.97 | | | 0.97 | 0.09 | | | | -0.17 | | 0.35 | | 0.48 | | 0.02 | | | -0.13 | | | 0.18 | | | 0.76 | | |
| Time | -0.31 | -0.38 | | -0.24 | | <0.01 | | -0.29 | | | | -0.86 | | | 0.28 | | | 0.31 | -0.22 | | | | -0.35 | | -0.08 | | <0.01 | | -0.33 | | | -0.41 | | | -0.26 | | | <0.01 | | |
| Debris index*Time | -0.20 | -0.36 | | -0.04 | | 0.02 | | -0.29 | | | | -1.39 | | | 0.81 | | | 0.60 | -0.42 | | | | -0.71 | | -0.14 | | <0.01 | | -0.08 | | | -0.34 | | | 0.19 | | | 0.57 | | |
| * Interaction effect (*p* <0.05)  Adjusted with age, sex, time (baseline or endline), interaction of each variable and time for all participants' analyses. Age variable was not adjusted for the age stratified model. | | | | | | | | | | | | | | | | | | | | | | | | | | | | | | | | | | | | | | | | |
| OHQOL: Oral health related quality of life, DMFT: Decayed, missing filled teeth | | | | | | | | | | | | | | | | | | | | | | | | | | | | | | | | | |  | |  |  | | |  |
| Time: Endline=1, Baseline =0 | | | | | | | | | | | | | | | | | | | | | | | | | | | | | | | | | |  | |  |  | | |  |

| 1. **Overall quality of life** | | | | | | | | |  | |  | | | |  |  | | | |  | | |  | | | |  |  | |  | |  | |  |  |
| --- | --- | --- | --- | --- | --- | --- | --- | --- | --- | --- | --- | --- | --- | --- | --- | --- | --- | --- | --- | --- | --- | --- | --- | --- | --- | --- | --- | --- | --- | --- | --- | --- | --- | --- | --- |
| Variables | All age groups | | | | | | 6-8 years old at baseline | | | | | | | 9-12 years old at baseline | | | | | | | | | | 13-15 years old at baseline | | | | | | | | | | | |
|  | Estimate | | 95% CI | | P Value | | Exp | | 95% CI | | | P Value | | Exp | | | | 95% CI | | | P Value | | | Exp | | 95% CI | | | | | P Value | | | | |
| OHQOL | -0.22 | -0.28 | | -0.16 | | <0.01 | -0.29 | -0.49 | | -0.09 | | | 0.01 | -0.22 | | | -0.31 | | -0.13 | | | <0.01 | | -0.21 | -0.29 | | | | -0.12 | | | | <0.01 | | |
| Time | -0.04 | -0.15 | | 0.07 | | 0.48 | -0.10 | -0.46 | | 0.26 | | | 0.58 | -0.05 | | | -0.21 | | 0.12 | | | 0.58 | | 0.04 | -0.15 | | | | 0.23 | | | | 0.70 | | |
| OHQOL*Time | 0.05 | -0.04 | | 0.13 | | 0.28 | 0.08 | -0.19 | | 0.36 | | | 0.55 | 0.05 | | | -0.08 | | 0.18 | | | 0.41 | | -0.01 | -0.16 | | | | 0.14 | | | | 0.92 | | |
|  |  |  | |  | |  |  |  | |  | | |  |  | | |  | |  | | |  | |  |  | | | |  | | | |  | | |
| Salivary pH | -0.03 | -0.26 | | 0.20 | | 0.79 | 0.48 | -0.23 | | 1.19 | | | 0.18 | -0.31 | | | -0.65 | | 0.03 | | | 0.08 | | 0.29 | -0.15 | | | | 0.73 | | | | 0.20 | | |
| Time | 0.13 | -0.14 | | 0.39 | | 0.36 | 0.50 | -0.30 | | 1.29 | | | 0.21 | -0.02 | | | -0.43 | | 0.40 | | | 0.93 | | 0.27 | -0.21 | | | | 0.74 | | | | 0.27 | | |
| Salivary pH*Time | -0.11 | -0.45 | | 0.23 | | 0.52 | -0.62 | -1.63 | | 0.39 | | | 0.22 | 0.08 | | | -0.44 | | 0.61 | | | 0.76 | | -0.29 | -0.90 | | | | 0.32 | | | | 0.34 | | |
|  |  |  | |  | |  |  |  | |  | | |  |  | | |  | |  | | |  | |  |  | | | |  | | | |  | | |
| Salivary flow | -0.06 | -0.11 | | -0.02 | | 0.01 | -0.15 | -0.25 | | -0.04 | | | 0.01 | -0.11 | | | -0.18 | | -0.03 | | | 0.01 | | 0.05 | -0.03 | | | | 0.13 | | | | 0.23 | | |
| Time | 0.01 | -0.03 | | 0.04 | | 0.73 | -0.04 | -0.11 | | 0.03 | | | 0.22 | 0.01 | | | -0.05 | | 0.06 | | | 0.81 | | 0.06 | <0.01 | | | | 0.12 | | | | 0.06 | | |
| Salivary flow*Time | 0.07 | 0.01 | | 0.13 | | 0.03 | 0.16 | 0.01 | | 0.32 | | | 0.04 | 0.09 | | | -0.02 | | 0.19 | | | 0.10 | | -0.04 | -0.15 | | | | 0.07 | | | | 0.49 | | |
|  |  |  | |  | |  |  |  | |  | | |  |  | | |  | |  | | |  | |  |  | | | |  | | | |  | | |
| DMFT/dmft | <-0.01 | -0.04 | | 0.03 | | 0.68 | -0.02 | -0.17 | | 0.12 | | | 0.75 | <0.01 | | | -0.05 | | 0.05 | | | 0.92 | | -0.02 | -0.08 | | | | 0.03 | | | | 0.42 | | |
| Time | 0.04 | <-0.01 | | 0.08 | | 0.05 | -0.02 | -0.22 | | 0.17 | | | 0.80 | 0.06 | | | <0.01 | | 0.12 | | | 0.06 | | 0.02 | -0.04 | | | | 0.08 | | | | 0.53 | | |
| DMFT/dmft*Time | <-0.01 | -0.05 | | 0.04 | | 0.93 | 0.04 | -0.16 | | 0.23 | | | 0.71 | -0.02 | | | -0.09 | | 0.06 | | | 0.67 | | 0.03 | -0.05 | | | | 0.11 | | | | 0.42 | | |
|  |  |  | |  | |  |  |  | |  | | |  |  | | |  | |  | | |  | |  |  | | | |  | | | |  | | |
| DMFT | 0.14 | -0.02 | | 0.05 | | 0.40 | -0.03 | -0.15 | | 0.09 | | | 0.58 | 0.05 | | | <0.01 | | 0.10 | | | 0.04 | | -0.01 | -0.07 | | | | 0.05 | | | | 0.83 | | |
| Time | 0.04 | 0.01 | | 0.07 | | 0.01 | -0.01 | -0.09 | | 0.06 | | | 0.69 | 0.08 | | | 0.04 | | 0.13 | | | <0.01 | | 0.03 | -0.03 | | | | 0.09 | | | | 0.27 | | |
| DMFT*Time | -0.01 | -0.05 | | 0.03 | | 0.67 | 0.07 | -0.08 | | 0.22 | | | 0.36 | -0.06 | | | -0.13 | | 0.01 | | | 0.08 | | 0.01 | -0.06 | | | | 0.09 | | | | 0.74 | | |
|  |  |  | |  | |  |  |  | |  | | |  |  | | |  | |  | | |  | |  |  | | | |  | | | |  | | |
| Debris index | <0.01 | -0.09 | | 0.09 | | 0.92 | -0.18 | -0.67 | | 0.31 | | | 0.47 | 0.01 | | | -0.18 | | 0.19 | | | 0.95 | | -0.21 | -0.29 | | | | -0.12 | | | | <0.01 | | |
| Time | 0.03 | -0.02 | | 0.08 | | 0.25 | -0.15 | -0.43 | | 0.13 | | | 0.29 | 0.02 | | | -0.08 | | 0.12 | | | 0.70 | | 0.04 | -0.16 | | | | 0.23 | | | | 0.70 | | |
| Debris index*Time | 0.04 | -0.07 | | 0.15 | | 0.46 | 0.38 | -0.16 | | 0.92 | | | 0.16 | 0.10 | | | -0.12 | | 0.31 | | | 0.37 | | -0.01 | -0.16 | | | | 0.14 | | | | 0.92 | | |
| * Interaction effect (*p* <0.05)  Adjusted with age, sex, time (baseline or endline), interaction of each variable and time for all participants' analyses. Age variable was not adjusted for the age stratified model. | | | | | | | | | | | | | | | | | | | | | | | | | | | | | |  | |  | |  |  |
| OHQOL: Oral health related quality of life, DMFT: Decayed, missing filled teeth | | | | | | | | |  | |  | | | |  |  | | | |  | | |  | | | |  |  | |  | |  | |  |  |
